# Exploring and Mapping Screening Tools for Cognitive Impairment and Traumatic Brain Injury in the Homelessness Context: A Scoping Review

**DOI:** 10.3390/ijerph20043440

**Published:** 2023-02-15

**Authors:** Erin M. Fearn-Smith, Justin Newton Scanlan, Nicola Hancock

**Affiliations:** Faculty of Medicine and Health, Centre for Disability Research and Policy, The University of Sydney, Camperdown, NSW 2050, Australia

**Keywords:** homelessness, cognitive impairment, traumatic brain injury, screening tools, case management, rehabilitation, recovery, supportive housing, social determinants of health

## Abstract

Cognitive impairment is common amongst people experiencing homelessness, yet cognitive screening and the collection of history of brain injury rarely features in homelessness service delivery practice. The purpose of this research was to scope and map strategies for screening for the potential presence of cognitive impairment or brain injury amongst people experiencing homelessness and identify instruments that could be administered by homelessness service staff to facilitate referral for formal diagnosis and appropriate support. A search was conducted across five databases, followed by a hand search from relevant systematic reviews. A total of 108 publications were included for analysis. Described in the literature were 151 instruments for measuring cognitive function and 8 instruments screening for history of brain injury. Tools that were described in more than two publications, screening for the potential presence of cognitive impairment or history of brain injury, were included for analysis. Of those regularly described, only three instruments measuring cognitive function and three measuring history of brain injury (all of which focused on traumatic brain injury (TBI)) may be administered by non-specialist assessors. The Trail Making Test (TMT) and the Ohio State University Traumatic Brain Injury Identification Method (OSU TBI-ID) are both potentially viable tools for supporting the identification of a likely cognitive impairment or TBI history in the homelessness service context. Further population-specific research and implementation science research is required to maximise the potential for practice application success.

## 1. Introduction

Homelessness is a growing global health concern, with increasing international commitment to addressing the problem [1]. The risk of becoming and remaining homeless can be significantly increased by a cognitive impairment [2], and the prevalence of cognitive impairment is much higher in homeless populations [3,4,5]. The primary causes of cognitive impairment in homeless populations have been attributed to developmental disability, psychotic illness, substance use, traumatic brain injury (TBI), and other neurological conditions [6]. Details pertaining to most of these issues are often routinely collected in homelessness service delivery, including disability status, psychiatric diagnoses, substance use behaviours, and diagnosed health conditions of people accessing the service. However, despite recommendations [5,6,7], collecting history of brain injury and screening for cognitive impairment are not standard practices in homelessness service delivery [8].

High rates of cognitive impairment [5,9,10,11], brain injury [12,13,14,15,16,17,18,19,20,21,22,23,24,25,26,27], or both [7,28,29] have been found in homeless populations. Failure to identify these issues amongst people experiencing homelessness affects other social determinants at the individual level, including poor housing, health, social, vocational, and justice outcomes for people in this cohort, places undue pressure on the homelessness service delivery system, and increases demand on emergency and other public service responses.

Cognitive impairments, resulting from brain injuries or other causes, are thought to play a role in limiting housing success [30,31]. Homeless populations have been found to have substantial deficits in memory, attention, learning, cognitive processing speed, and executive function [3,32], and this has been associated with functional limitations, including poor problem solving and independent living skills, increased risk taking and interpersonal conflict, and reduced community participation and income [33,34,35,36].

A scoping review conducted by Stone et al. [2] highlights that in addition to increased rates of cognitive impairment among people who are homeless, risks associated with becoming or remaining homeless are exacerbated by socioeconomic factors. As a group who are often less engaged with employment, education, and regular planned and preventive health and welfare services [15,29,37], poorer health and housing outcomes are common [3,4]. While improvements in cognitive function have been observed upon resolution of homelessness [38,39], ongoing support needs have been found amongst those with a diagnosed cognitive impairment [40]. Securing appropriate support services to coincide with housing acquisition is, therefore, likely to improve housing sustainability for people exiting homelessness with a cognitive impairment.

In a study that looked at case manager estimations of cognitive function amongst their homeless clients, Vella [41] found that prior to screening, cognitive and functional capacity of people accessing homelessness services was overestimated by service staff, resulting in unrealistic expectations of independent living success. This may lead to inadequate referrals for specialist services and housing choices that are not sustainable without support, and in turn may be associated with repeat use of homelessness services [41]. Another study found that staff both over- and underestimate homeless clients’ cognitive capacity and that homelessness service providers care for substantial numbers of people with memory problems, cognitive decline, and dementia who are not receiving the necessary specialist services [42]. This increases pressure on the homelessness service system and reduces overall capacity to deliver services to people without complex needs. This increased demand may, therefore, contribute to significant numbers of people being unable to access timely services, as has been reported in the US [43], Canada [44], and Australia [45].

In addition to poor individual outcomes, inadequate engagement with planned health and welfare services, and inefficient utilisation of the homelessness service system, long-term homelessness is associated with increased use of emergency and other public resources. People experiencing homelessness, cognitive impairment, and TBI are more likely to have contact with police, fire service and paramedics, increased presentations to emergency departments, greater frequency of hospital admissions, high use of emergency housing and welfare services, and are more likely to be in contact with state justice and child protection [24,46,47]. A similar profile exists for people with cognitive impairment who are housed with inadequate support [48]. As such, there is a strong economic argument for disrupting the cycle of homelessness through the prompt identification of cognitive impairment and brain injury.

Formal assessment and diagnosis are required to open pathways to appropriate support for people with a cognitive impairment. When facilitating this, health professionals are infrequently readily available either to homelessness services, which are often under resourced, or to homeless cohorts more generally, who are often disengaged from services. Behaviour associated with brain injury or cognitive impairment is very often attributed to substance use, mental health, or ‘difficult’ behaviour. The introduction of efficient screening for cognitive impairment and brain injury, as a brief indicator of a potential issue, as opposed to a clinical examination, may offer an efficient prompt for formal assessment and diagnosis, facilitate appropriate service delivery, and attract more suitable funding streams. This would support long-term and repeat users of homelessness services to access service environments that have capability and capacity to understand the impacts of cognitive impairment or brain injury on functional impairments, as well as add new capacity to homelessness programs, and reduce the unplanned use of other public resources. 

Despite this opportunity for improved health outcomes and resource allocation, routine screening for cognitive impairment and brain injury does not feature in homelessness service delivery practice [11,22,49]. Research in this area has asserted the importance of identifying cognitive impairment and TBI [7,8,9,14,50,51,52,53], and provided helpful recommendations for clinicians in identifying cognitive impairment utilising abbreviated batteries more suited to a homeless client population [54,55], as well as guidelines for working with homeless people with an identified cognitive impairment or brain injury [11,51,56]. However, given that a great number of homelessness programs are operated by non-government organisations [57,58,59,60], a gap exists for people in this population who are being supported exclusively in non-clinical homelessness services by non-clinical staff. A system for identifying a likely cognitive impairment or brain injury to prompt referral for formal assessment is, therefore, required. This would maximise opportunities for this population to be appropriately diagnosed, treated, and supported in healthcare and disability service environments. 

With a view to improving referral pathways in practice, this study aimed to explore instruments described in the peer-reviewed literature that have been used to identify cognitive impairment or brain injury within the homeless population, specifically. Existing reviews have explored prevalence and instruments that assess either cognitive function or history of brain injury [2,26,27]. These studies found that prevalence of impairment is high in this population, great variation in aetiology exists, and this population experience comorbid health problems and have compromised functional outcomes. They also demonstrate that prevalence studies are overrepresented in the literature and that qualitative studies capturing the voices of this population are underrepresented.

Building on those works, the objective of this research was to scope and map what is contained in the literature and specifically identify who was being screened and where, the instruments regularly employed to screen for the potential presence of cognitive impairment and brain injury amongst people experiencing homelessness, and instruments that are potentially viable for implementation in homelessness service delivery practice. Considerations in viability for practical application included:

- Unrestricted in terms of qualifications of the person administering the screening tool.
- Economical in terms of cost and time resources required.
- Considerations of acceptability to assessors and those being assessed.
- Sensitive and specific to identifying a brain injury or cognitive impairment amongst people accessing homelessness services.

## 2. Materials and Methods

The design of this study is a scoping review, selected as an effective method for exploratory research into a complex topic. The purpose of the research is aligned with the common reasons outlined for undertaking this type of study [61]: to identify and map the nature and extent of research available in the literature regarding the identification of potential cognitive impairments or brain injury amongst people who are experiencing homelessness. The applied methodology reflects the framework published by Arksey and O’Malley [61], supported by JBI guidance [62], and reported following the Preferred Reporting Items for Systematic Reviews and Meta-Analyses (PRISMA) guidelines [63].

### 2.1. Identification of the Research Questions

This scoping review was designed with the intention of answering the following research questions:

- What tools or strategies have been employed in the literature for identifying cognitive impairment or brain injury amongst people who are experiencing homelessness?
- Who are the populations being assessed in the literature, and under what circumstances?
- Are there tools that can be administered in non-clinical homelessness service environments, by non-specialist staff, that are sensitive to identifying a likely cognitive impairment or brain injury?

### 2.2. Identification of Relevant Literature

The purpose of a scoping study is to engage in a thorough review of the available literature. Records were generated through a systematic database search and hand searching through relevant systematic reviews that were generated through the database search. 

#### 2.2.1. Search Terms

As a scoping review, this study employed the PCC mnemonic (Population; Concept; Context) described by the Joanna Briggs Institute (JBI) [64,65] for developing terms to be applied in search strategy design and record screening for scoping reviews. This study considered all types of peer reviewed research that focused on the following:

- Population: people experiencing or at risk of homelessness.
- Concept: identifying cognitive impairment or brain injury.
- Context: assessment, screening, or measurement.

#### 2.2.2. Database Search

A preliminary database search using 20 search terms was conducted to generate relevant key words to develop a search strategy, resulting in a total of 57 terms applied to the search. In Week 3 of January 2021, database searches were conducted in CINAHL, Embase, Medline, PsycInfo, PsycTests, Scopus, and Web of Science. Medical Subject Heading (MeSH) terms were applied; however, of the mapped terms related to the concept, specific diagnoses were not included, and mapped terms related to the context did not include specific tools. Terms related to the population were combined with ‘OR’, as were terms related to the concept and the context. The collated results of these three searches were combined with ‘AND’. The search strategy is available in Appendix A Table A1 ‘Search strategy’.

Database search results were exported to Endnote, and records were exported to Covidence for screening against inclusion criteria. Relevant studies were mapped in Microsoft Excel.

#### 2.2.3. Hand Search

Although systematic reviews were not included in the study, further relevant publications were sourced by hand searching the systematic reviews that were generated from the database search.

### 2.3. Selection of Studies

Literature was screened by two reviewers using online Covidence software [66], and sources were assessed, by title and abstract, and then full text, against the inclusion criteria (see Table 1. Inclusion and exclusion criteria). Papers for inclusion described screening for any potential impairment in cognitive functioning or history of brain injury amongst people who were homeless or at risk of returning to homelessness. As this study seeks to identify possible tools for pilot implementation in practice settings, studies describing only paediatric participants were excluded. This decision was made both because instruments screening for paediatric cognitive impairment often cannot be applied with adult populations (and vice versa), and because unaccompanied children under 16 years are usually intended to be supported through state authorities rather than homelessness services. Acknowledging that family interventions are common in homelessness service delivery, and unaccompanied minors are often neglected through state-based out-of-home care structures, investigation in this area will be recommended for future studies. 

Covidence captured any conflicts between reviewers regarding inclusion or exclusion (such as when studies included both homeless and housed populations or participants both over and under the age of 16), and consensus was reached through discussion and mutual agreement to include all studies that specifically reported results reflecting the population of interest.

#### 2.3.1. Title and Abstract Screening

Items were first assessed by title and abstract, excluding irrelevant publications. Titles with no available abstract were individually searched using the University of Sydney Cross-Search tool. Items that were not able to be located locally were ordered through inter-library loans.

#### 2.3.2. Full Text Screening

The remaining items were screened using the full text and assessed against the inclusion criteria by both reviewers. Relevant literature reviews that were generated through the database search were excluded from the study but included for hand searching potential additional papers.

### 2.4. Data Mapping

Data were mapped into the following categories: study details, sampling, instruments applied, prevalence, administered by, administration time, and acceptability to consumers and assessors. A complete data map is available as a Supplementary File (Table S1: Data Map).

### 2.5. Collation, Summary, and Report of Results

A PRISMA [63] flowchart was employed for presentation of the selection and exclusion of studies. A summary of findings from data mapping is presented in tables and discussed in relation to the research questions.

## 3. Results

### 3.1. Selection of Studies

**Figure 1:**
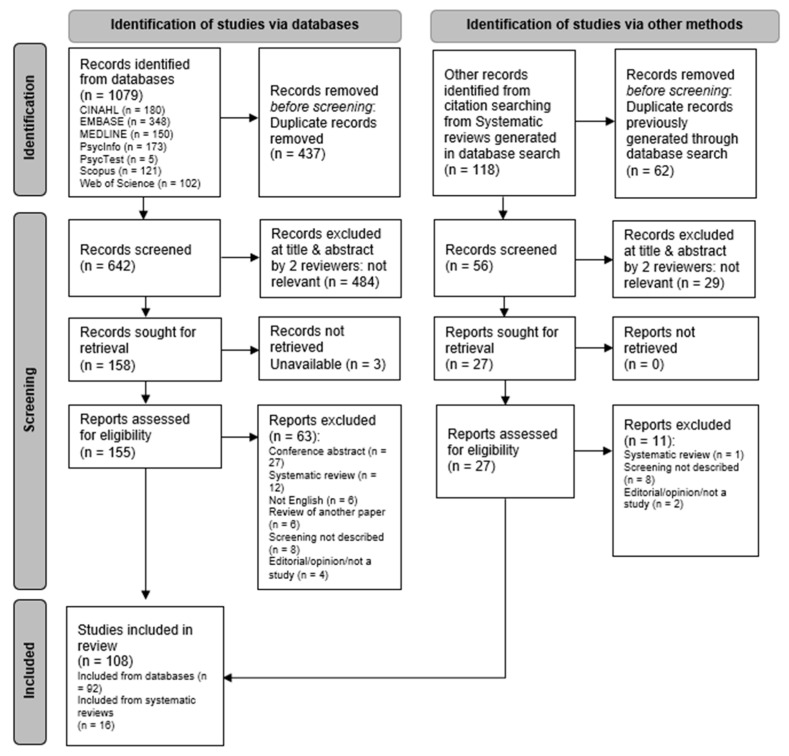
PRISMA flow diagram [63].

The PRISMA diagram in Figure 1 demonstrates the data identification and screening process that informed the selection of studies. A total of 108 publications were included in the final review (available as Table A2 in Appendix B ‘Literature chart’).

### 3.2. Extracting and Charting

The literature was mapped against fields recommended by the JBI (2017): author(s), year of publication, source origin/country of origin, study population and sample size, study setting, instruments applied, administration, screening outcomes, duration of assessment, how outcomes are measured, and key findings that relate to the review questions.

Included papers ranged in publication date between 1990 and 2021, and included 12 dissertation publications and 96 peer-reviewed articles. Articles spanned 66 journals, with a median of 1 publication per journal: 48 journals published 1 study, 13 journals published 2 studies, and only 5 journals published 3 or more studies in this area of research. 

#### 3.2.1. Populations and Contexts

Literature summary features are shown in Table 2. Of the focus populations across the studies, though there was great variability in participant age, setting, and co-morbid diagnoses, the majority of people represented in the literature were men in Western countries. Almost three-quarters of the papers reported studies conducted in North America (USA, n = 56; Canada, n = 23), and most of the remainder reflected populations in Western, English-speaking countries. Men were most heavily represented in participant populations, with 99 of the 108 papers comprising more than 50% male participants.

All studies included screening people experiencing homelessness; however, not all studies were conducted exclusively with this population, or in settings exclusive to this population. Ninety-four studies were conducted in settings exclusive to people who are homeless: crisis accommodation or shelter environments (n = 42), generalist homelessness or outreach support settings (n = 31), post-crisis housing support (n = 11), and health settings for people who are homeless (n = 10). Fourteen studies were conducted in other health or community settings that service both people who are and who are not homeless, including general health or hospital settings (n = 7), mental health or substance use treatment programs (n = 5), and diverse community or social welfare settings (n = 2). The focus populations also varied across the literature, with just over half of the study populations being homeless adults (40% mixed, 9% men only, 4% women only), and the remaining studies focusing on populations with additional criteria for inclusion, such as age, cultural group, mental health status, substance use, veterans, or co-/multimorbidity (see Table 3). The majority of studies focused on measuring cognitive function (n = 87), though 31 of those papers also reported the presence of brain injury amongst participants. The remaining 21 studies focused exclusively on screening for brain injury. 

A number of issues relevant to this group were regularly reported across the literature. Participant data were often inclusive of issues pertaining to substance use (n = 85), schizophrenia or other psychotic illness (n = 50), diagnosed mental illness (specified, other than psychotic illness) or undefined mental illness (n = 82), length of homelessness considered (n = 56), frailty or health conditions (n = 28), veteran (n = 19), developmental disability (n = 15), adverse childhood experiences (n = 11), history of incarceration (n = 10), and domestic and family violence (n = 4).

#### 3.2.2. Screening Instruments

Across the 108 publications, a total of 158 separate instruments were described, including individual components of a single assessment tool and various versions of the same instrument component (see Appendix C, Table A3 ‘Complete list of screening instruments’). Of the 158 instruments, 151 were cognitive screens and 7 were TBI screens.

After grouping together identical instruments (including unchanged components of updated assessment tools), reviewers found 17 instruments measuring cognitive function and 3 screens for brain injury (all of which focused only on screening for TBI) that appeared in more than two publications, and that were used at least twice to explicitly screen for the presence or absence of cognitive impairment or TBI, as opposed to reporting a collated result to provide population-based performance data (see Table 3). 

Only 65% of those studies included details of who was administering the assessments, and of those, sometimes specialist training was implicit in the description (e.g., ‘psychiatrist’) and sometimes it was unclear (e.g., ‘research assistant’ or ‘case manager’). Further investigation was undertaken to determine whether specialist qualifications were required to administer the instruments, by first searching Pearson Assessments [67] or PAR Inc. [68] for qualification grade, and if unavailable on these platforms, through developer websites. This process determined that 14 of the 17 cognitive screening tools require specialist qualifications to administer them (psychologist, occupational therapist, or ‘health professional’), whilst the remaining 3, as well as the 3 TBI screens, are not restricted. Each of the unrestricted tools are also free to access. 

#### 3.2.3. Instruments Unrestricted by Qualification

To consider potential viability for practice implementation, the features of unrestricted screening approaches were mapped. The most commonly employed instrument for assessing cognitive function was the Mini Mental State Examination (MMSE) [69], followed by the Trail Making Test (TMT) [70] Parts A (TMT-A) and B (TMT-B). The most common approach to determining the presence of a TBI was a single or series of questions regarding history of head trauma, followed by The Ohio State University TBI Identification Method (OSU TBI-ID) [71] and the TBI-4 [72].

There was great variability between studies regarding the measured prevalence of cognitive impairment or TBI in the participant population. Time to administer was usually reported as a collated total time to administer a battery rather than time for the specific component. Details about sensitivity, specificity, and acceptability to consumers and assessors were rarely reported (see Table 4). 

#### 3.2.4. Acceptability of Instrument to Assessors and Consumers

Consideration was given to the acceptability of tools in a very small number of papers. In total, 4 of the 108 included publications considered the acceptability of the assessment process to both assessors and consumers, 1 further study considered acceptability to consumers only, and another considered acceptability to assessors only. 

Of the screening tools that appeared regularly in the literature and can be employed by non-specialist assessors, the acceptability to both consumers and assessors was considered for the MMSE and Trail-Making test Parts A and B in one single study [54], and the acceptability for assessors was considered for the OSU TBI-ID in one study [22]. Considerations included: time required; incorporation into routine appointments; both assessor and client preferences for time of day, with consideration of substance use or medication effects; demands of the assessment influencing willingness of consumer engagement; practicality in administration and interpretation; and the cost-effectiveness of the tool.

#### 3.2.5. Sensitivity and Specificity

Sensitivity and specificity were rarely described; consideration of sensitivity and specificity of the unrestricted instruments was described, or implied, in four studies. Under circumstances where they were described, it was unclear whether this reflected validated administration with homeless populations.

##### Cognitive Screens

Mini Mental State Examination (MMSE). Gash [73] described strong sensitivity and specificity of the MMSE in identifying moderate or severe cases of dementia only. Whilst Gonzalez et al. [54] did not explicitly describe sensitivity and specificity, this study did compare MMSE results with results from a neuropsychological test battery and found that although 80% of the population were found to have a cognitive impairment using the test battery, the MMSE measured prevalence of only 35%. No studies were located that specifically reported on studies evaluating specificity of the MMSE with identifying cognitive impairments within homeless populations.

Trail Making Test—Part A (TMT-A). Sensitivity and specificity of TMT-A was not described in the included literature.

Trail Making Test—Part B (TMT-B). Though sensitivity and specificity of TMT-B was not explicitly described in the included literature, Gonzalez [54] found a close correlation between results of TMT-B and the results of an abbreviated neuropsychological test battery.

Similar to the MMSE, no studies were located that explored the specificity of TMT-A or TMT-B within homeless populations.

##### TBI Screens

OSU TBI-ID. Lafferty [17] described being unable to locate information in the literature pertaining to the sensitivity and specificity of this tool; however, Russell [20] described the OSU TBI-ID as the ‘gold standard’ in identifying TBI.

TBI-4. This is an abbreviated, adapted version of the OSU TBI-ID implemented as a preference to single-question approaches to determining TBI history [72]. Russell et al. [20] found this tool to underestimate the prevalence of TBI, compared to the OSU TBI-ID.

Single or series of questions, e.g., “Have you ever had a blow to the head?”. As this was not consistently reported and does not describe a specific measure, no assessment of sensitivity or specificity applies to this method.

## 4. Discussion

Routine screening for brain injury and cognitive impairment is likely to improve referrals for earlier diagnosis and access to health and disability services, and the results of this study have important implications for practice in homelessness service delivery. Unlike details pertaining to mental health, substance use, and other known diagnoses, screens for cognitive dysfunction or history of brain injury in homeless populations are not routinely taken. Under circumstances where screening for brain injury or cognitive impairment is undertaken, measures are mostly collected for the purposes of research, such as to estimate prevalence of injury or impairment within this population. Most instruments employed in the literature require specialist training, and those that do not are limited in explicit descriptions of consideration for the service user or assessor experience, sensitivity and specificity for the identification of brain injury or cognitive impairment, and of whether the instrument has been validated with homeless populations.

With such limited research in this area, there was little consistency in focus or approach across the included literature. Substantial variability existed regarding participant gender, age, presence of co-morbidity, or type of service engagement. Some studies included only participants with a known brain injury or cognitive impairment, and some studies excluded those with existing diagnoses, particularly dementia or developmental disability. Assessment purposes varied but were rarely in the context of routine screening in service delivery, and when screening was routine, it was in a clinical context. Administrator profiles were inconsistently reported. When the assessor was reported, this was almost invariably a trained researcher or clinician. Assessment settings were also diverse. Homelessness-specific services included street outreach, crisis shelters, homeless health services, veterans homeless services, and post-homeless supportive housing programs, whilst non-homelessness-specific settings included health clinics, hospitals, community-based mental health services, detox and rehabilitation programs, community centres, and veterans services. This leaves little room for meaningful comparisons between the cohorts, the assessors, and the settings for screening people experiencing or at risk of homelessness for cognitive impairment or brain injury.

Of the potentially viable screens, whether sensitive or specific to identifying cognitive impairment or brain injury, none were validated in homeless populations [2,3,6,26,27,71,74,75]. Probably due to its unrestricted access and common administration in this area of research, the most frequently employed assessment of cognitive function in homeless populations was the MMSE. Although this tool is a reliable and validated [76] measure for the presence of dementia, limited effectiveness for identifying other cognitive deficits in homeless populations compared to other measures has been observed [54,77]. Of the remaining cognitive screens, parts A and B of the Trail Making Test (TMT) are also sensitive to identifying the presence of dementia [78] and other cognitive impairment [79], with greater cognitive demand evidenced with Part B [80,81]. Though this instrument has been described as a reliable indicator of cognitive impairment [79] and is evidenced to have results that are closely correlated with more comprehensive neurocognitive testing in people who are homeless [54], findings in other studies [32] have been inconsistent. However, TMT is brief, free to access, and has increased sensitivity with the inclusion of validated age- and education-adjusted cut-off scores [78,82]. It is also available in both pen-and-paper and digital formats, with possible additional cognitive processes that can be measured with digital formats of the test [83].

The most common screen for a brain injury was a single or series of questions regarding history of head trauma; however, this does not reflect a uniform approach to assessment and limits positive screens to those who are already aware that they have a brain injury and only focuses on TBI rather than the broader range of potential brain injury. Of the remaining TBI screens, the TBI-4 is a validated predictor of hospital admission in veteran populations [84], though less effective than the OSU TBI-ID method in identifying history of TBI amongst people who are homeless [20]. The OSU TBI-ID is a brief, valid, and reliable screen for history of TBI [74,85] that was developed based on TBI surveillance recommendations made by the Center for Disease Control and Prevention (CDC) [71]. Reporting errors are limited through the inclusion of head and neck injuries, length (if relevant) of loss of consciousness, number of injuries, age of first injury, and experience of dizziness and memory loss. Russell et al. [20] described the OSU TBI-ID as the ‘gold standard’ of TBI identification, and the instrument has been validated with military personnel, veterans, individuals with substance use, and prisoners or those with a history of incarceration [74,75,86]. The papers employing the OSU TBI-ID in this study did not specify whether they used the short, clinical, or research version of the instrument; however, the developers [71] describe the short version as being administered in 5 min. The OSU TBI-ID is also free to access.

Whilst engaging non-specialist staff in the screening process offers the promise of new referral prompts and better health and housing outcomes, potential consequences for the administration of cognitive screening tools by non-trained clinicians must also be considered. Application of screening in service settings carries the risk of being introduced as a barrier to service access, with identified scores required for inclusion or exclusion for service delivery. This could result in people with perceived impairment being excluded from services, remaining homeless until they decompensate and need institutionalised care, or experiencing early mortality. Recommendations for cognitive screening protocols in service delivery environments, therefore, include clear differentiation between screening and assessment, to ensure that non-specialist staff are clear that they are not undertaking assessment, but rather seeking to provide screening results in referrals for formal assessment. A matrix or decision tree to explicitly facilitate referral to appropriate services in the local context may also support meaningful and consistent referral outcomes.

A few limitations of this review need acknowledgement. Because of the limited body of literature in this area, when selecting papers, no discrimination was made between types of homeless populations, including definitions of homelessness and legislative or policy contexts. There were also several publications that reported data from the same studies, potentially over-representing the application of described instruments and populations receiving services. Finally, despite the diversity of search terms included, given the inconsistency of language and keywords in the literature, some relevant studies may not have been included.

## 5. Conclusions

Of the unrestricted instruments regularly reported in the homelessness literature, none offer an ideal screening tool for non-clinical homelessness service contexts to use to reliably identify a cognitive impairment within this population. However, the viability and value of implementing TMT in service delivery practice (either both parts, or Part-B only), as a fairly robust and time-effective screen for cognitive impairment with homeless populations, should be further explored. Similarly, the OSU TBI-ID offers a promising instrument for the identification of TBI, despite not capturing other forms of brain injury. The estimated ten minutes for completion of these two assessments may support routine implementation viability within homelessness service sites.

Further investigation is required in the pursuit for successful routine screening for cognitive impairment and TBI in homeless populations. Consultation with people who either work in or access homelessness services regarding facilitators and barriers to routine practice implementation is recommended in the development of a screening protocol that is acceptable to users. Potentially viable instruments are recommended to be validated with this population as well as further research exploring appropriate screening instruments for history of brain injuries other than TBI. As the application of brief screening instruments alone may not capture all those benefiting from more comprehensive assessment and support, the identification of other common factors amongst this cohort may be helpful in enhancing screening protocols and providing further indicators of potential impairment and referral needs in this group. The evaluation of application in service delivery within specific contexts will also determine the impact of screening in different services (e.g., shelter versus health centres) and different geographical locations. Finally, undertaking similar research to identify cognitive impairment and brain injury in paediatric populations experiencing homelessness may facilitate early intervention and prevent long-term experiences of homelessness.

## Figures and Tables

**Table 1:** Inclusion and exclusion criteria.

Inclusion Criteria	Exclusion Criteria
Screening for identification of any potential impairment in cognitive functioning or brain injury amongst people aged 16+ years who are homeless (or exiting homelessness, or at risk of returning to homelessness) described. Peer-reviewed studies (incl. dissertations) Primary research Written in English	Describe only populations aged <16 years Conference abstracts or poster presentations Editorials, opinions, reports, or publications that are not peer-reviewed primary research studies Unavailable in full text Systematic reviews: *reviewers to hand search for potentially suitable papers*

**Table 2:** Literature Summary Features.

Summary Features	n (%)
**Country**	
USA	56 (52%)
Canada	23 (21%)
UK	12 (11%)
Australia	4 (4%)
Japan	4 (4%)
Other ^^^	9 (8%)
**Screen**	
Cognitive function only	56 (52%)
Presence of brain injury only	21 (19%)
Both described	31 (29%)
**Instruments**	
Single measure	38 (35%)
Battery	70 (65%)
**Study Populations**	
Adults (general n = 43; Men only n = 10; Women only n = 4)	57 (53%)
Mental Health (MH)/Substance Use Disorder (SUD)	20 (19%)
Older People (50+)	11 (10%)
Veterans (General n = 5; Veterans with MH/SUD n = 3)	8 (7%)
Young People (16–24)	6 (6%)
Co- or Multi-morbidity	4 (4%)
CALD/BME	1 (1%)
Indigenous	1 (1%)
**Study Participants**	
50%+ male participants	99 (92%)
50%+ female participants	7 (6%)
Unknown/not specified	2 (2%)
**Study Settings**	
Homeless-specific settings	94 (87%)
Other community settings	14 (13%)

Notes. n = number; ^^^ incl. Brazil, Ecuador, Germany, Hong Kong, Israel, Mozambique, Spain, and Netherlands.

**Table 3:** Instruments regularly screening for the presence of brain injury or cognitive impairment.

Instrument	Administration Restriction	Purchase/Administration Cost
**Cognitive screening instruments**		
Allen Cognitive Level Screen-2000/ACLS-5	Specialist ^a^	Yes
Connors’ Continuous Performance Test (CPT-II)	Specialist	Yes
Delis–Kaplan Executive Function System (D-KEFS) Tower	Specialist	Yes
Delis–Kaplan Executive Function System (D-KEFS) Trail Making Number-Letter Switching	Specialist	Yes
Delis–Kaplan Executive Function System (D-KEFS) Verbal Fluency-Category Switching	Specialist	Yes
Hopkins Verbal Learning Test-R (HVLT-R)	Specialist	Yes
Montreal Cognitive Assessment (MoCA)	Specialist ^a^	Yes
Rey Complex Figure Test (RCFT) copy trial	Specialist	Yes
Rey Complex Figure Test (RCFT) delayed recall	Specialist	Yes
Wechsler Abbreviated Scale of Intelligence (WASI)	Specialist	Yes
WASI Vocabulary subtest	Specialist	Yes
WAIS-R/WAISIII/WAIS-IV Digit Span	Specialist	Yes
WAIS-R Digit Symbol/WAIS-III Digit-Symbol coding/WAIS-IV Coding	Specialist	Yes
Wechsler Test of Adult Reading (WTAR)	Specialist	Yes
Mini Mental State Examination (MMSE)	None	No
Trail Making Test-Part A	None	No
Trail Making Test-Part B	None	No
**TBI Screening instruments**		
OSU TBI-ID	None	No
TBI-4	None	No
Single or series of questions, e.g., “Have you ever had a blow to the head?”	None	No

Note. ^a^ No restriction; however, developer recommends restriction to health professionals.

**Table 4:** Key features of unrestricted screens.

	Cognition Screens			TBI Screens		
	Mini Mental State Examination (MMSE)	Trail Making Test-Part A (TMT-A)	Trail Making Test-Part B (TMT-B)	Ohio State University Traumatic Brain Injury Identification Method (OSU TBI-ID)	TBI-4	Single/Series of Questions, e.g., “Have You Ever Had a Blow to the Head?”
**Papers Describing the Measure**						
n	20	8	11	10	3	14
**Positive Screen**						
median	24%	44%	38%	90%	73%	58%
mean	24%	50%	48%	82%	73%	55%
range	0–78%	27–80%	25–80%	53–91%	59–87%	9–84%
*(collated result) ^1^*	*(5%)*	*(63%)*	*(45%)*	*(20%)*	*(33%)*	*(0%)*
**Time to Administer**						
range	? ^1^	? ^1^	? ^1^	? ^1^	? ^1^	? ^1^
*(collated result) ^1^*	*(90%)*	*(88%)*	*(82%)*	*(100%)*	*(100%)*	*(86%)*
**Client Acceptability Considered**						
times described	1	1	1	0	0	0
**Assessor Acceptability Considered**						
times described	1	1	1	1	0	0
**Sensitivity and Specificity of Instrument Described**						
times described	1 ^2^	0	1 ^2^	1 ^3^	1 ^3^	NA

Notes. n = number; ^1^ studies presenting a result that is a global score or reflects total time for a test battery; ^2^ described explicitly in one study; however, sensitivity also described in a second study, comparing MMSE results and TMT-B to neuropsychological battery results; ^3^ one study explicitly mentions sensitivity and specificity, in the context of being unable to find this information.

## Data Availability

The data presented in this study are not publicly available.

## Supplementary Materials

The following supporting information can be downloaded at: https://www.mdpi.com/article/10.3390/ijerph20043440/s1, Table S1: Data Map.

## Appendix A

**Table A1:** Search Strategy.

*Cognitive Impairment or ABI*	*Screening, Interview or Assessment*	*People Who Are Homeless*
Keyword search terms	Keyword search terms	Keyword search terms
(1) brain damage *	(18) screen *	(42) homeless *
(2) brain disease *	(19) health screen *	(43) rough sleep *
(3) brain disorder *	(20) screen * tool *	(44) sleep * rough *
(4) brain injur *	(21) dementia screen *	(45) unsheltered
(5) acquire * brain injur *	(22) assessment *	(46) vulnerabl * hous *
(6) traumatic brain injur *	(23) health assessment *	(47) crisis hous *
(7) head injur *	(24) assessment tool *	(48) crisis accommodation *
(8) brain tumor *	(25) dementia assessment *	(49) transition * hous *
(9) cogniti * impair *	(26) cogniti * function * assessment *	(50) transition * accommodat *
(10) cogniti * dysfunction *	(27) neuropsych * assessment *	(51) halfway hous *
(11) cogniti * disorder *	(28) executive function * assessment *	(52) homeless patient *
(12) cogniti * disabilit *	(29) case manage * assessment *	(53) marginal * hous *
(13) dementia	(30) intake assessment *	(54) homeless * program *
(14) cogniti * defici *	(31) cogniti * function * test *	(55) homeless * service *
(15) cogniti * defect *	(32) executive function * test *	(56) homeless * support *
(16) alcohol related brain injur *	(33) neuropsych * test *	(57) specialist homeless * service *
(17) COMBINE WITH OR	(34) dementia test *	(58) going home staying home
	(35) interview tool *	(59) Supported Accommodation Assistance Program
	(36) intake interview *	(60) COMBINE WITH OR
	(37) case manage * interview *	
	(38) dementia interview *	
	(39) instrument *	
	(40) assessment instrument *	
	(41) COMBINE WITH OR	
(61) COMBINE (17), (41) & (60) WITH AND		

Note. * is the truncation operator applied to search terms.

## Appendix B

**Table A2:** Literature chart.

Publications Included in Study	Sample Size	Mean Age	% Male	Population Focus	Setting	Country	Instruments
Adams, C. E., Pantelis, C., Duke, P. J., & Barnes, T. R. (1996). Psychopathology, social and cognitive functioning in a hostel for homeless women. *British Journal of Psychiatry*, *168*(1), 82–86. [87]	64	44	0.0%	women only	Crisis/shelter	UK	- Mini Mental State Examination (MMSE) - Raven’s Standard Progressive Matrices
Adams, S. M. (2009). *Neuropsychological functioning and attrition rates in outpatient substance dependence treatment* ProQuest Dissertations Publishing]. [55]	68	45	100.0%	SUD	Crisis/shelter	USA	- Connors’ Continuous Performance Test (CPT-II) - Delis–Kaplan Executive Function System (D-KEFS) Colour Word Inhibition Test - Delis–Kaplan Executive Function System (D-KEFS) Tower - Delis–Kaplan Executive Function System (D-KEFS) Trail Making motor speed (test #5) - Delis–Kaplan Executive Function System (D-KEFS) Trail Making Number-Letter Switching - Delis–Kaplan Executive Function System (D-KEFS) Verbal Fluency-Category Fluency - Delis–Kaplan Executive Function System (D-KEFS) Verbal Fluency-Category Switching - Delis–Kaplan Executive Function System (D-KEFS) Verbal Fluency-Letter Fluency - Delis–Kaplan Executive Function System (D-KEFS) Trail Making Visual Scanning - Delis–Kaplan Executive Function System (D-KEFS) Trail Making Number Sequencing - Delis–Kaplan Executive Function System (D-KEFS) Trail Making Letter Sequencing - Wechsler Abbreviated Scale of Intelligence (WASI)
Adshead, C. D., Norman, A., & Holloway, M. (2019). The inter-relationship between acquired brain injury, substance use and homelessness; the impact of adverse childhood experiences: an interpretative phenomenological analysis study. *Disability and rehabilitation*, 1–13. [50]	8	41	50.0%	co-/multi-morbidity	Health service for homeless people—homeless/A & D service	UK	- Self-report of cognitive function and memory—interview only - Brain Injury Screening Index (BISI)
Andersen, J., Kot, N., Ennis, N., Colantonio, A., Ouchterlony, D., Cusimano, M. D., & Topolovec-Vranic, J. (2014). Traumatic brain injury and cognitive impairment in men who are homeless. *Disability and rehabilitation*, *36*(26), 2210–2215. [7]	34	58.8	100.0%	men only	Homeless service with accommodation	Canada	- Repeatable Battery for the Assessment of Neuropsychological Status (RBANS) - Brain Injury Screening Questionnaire (BISQ)
Bacciardi. S., Maremmani, A. G. I., Nikoo, N., Cambioli, L., Shutz, C., Jang, K., Krausz, M. (2017). Is bipolar disorder associated with tramautic brain injury in the homeless? *Riv Psichiatr 52*(1), 40–46. [88]	416	39.93	94.5%	co-/multi-morbidity	Housing support program to people at risk/formerly homeless	Canada	- Single or series of questions, e.g., “Have you ever had a blow to the head?”
Barak, Y., & Cohen, A. (2003). Characterizing the elderly homeless: a 10-year study in Israel. *Archives of Gerontology & Geriatrics*, *37*(2), 147–155. [89]	98	71.7	95.9%	older people	Homeless support without accommodation—homelessness street outreach	Israel	- Mini Mental State Examination (MMSE)
Barnes, S. M., Russell, L. M., Hostetter, T. A., Forster, J. E., Devore, M. D., & Brenner, L. A. (2015). Characteristics of Traumatic Brain Injuries Sustained Among Veterans Seeking Homeless Services. *Journal of Health Care for the Poor & Underserved*, *26*(1), 92–105. [12]	229	51.8	96.1%	Vets	General, mixed or unspecified homelessness support—homeless service (veterans)	USA	- OSU TBI-ID
Barone, C., Yamamoto, A., Richardson, C. G., Zivanovic, R., Lin, D., & Mathias, S. (2019). Examining patterns of cognitive impairment among homeless and precariously housed urban youth. *Journal of Adolescence*, *72*, 64–69. [9]	44	21.5	62.8%	young people	Health service for homeless people—homeless + MH	Canada	- California Verbal Learning Test (CVLT) - Controlled Oral Word Association Test (COWAT) (FAS) - Hopkins Verbal Learning Test-R (HVLT-R) - Repeatable Battery for the Assessment of Neuropsychological Status (RBANS) - Trail Making Test-Part A - Trail Making Test-Part B - Wechsler Adult Intelligence Scale 4thEd (WAIS-IV) - Wechsler Memory Scale 4thEd (WMS-IV) - Wide Range Achievement Test 4thEd (WRAT-IV) - Wisconsin Card Sorting Test (WCST)
Benda, B. B. (2004). *Gender Differences in the Rehospitalization of Substance Abusers Among Homeless Military Veterans.* Journal of Drug Issues. Vol.34(4), 2004, pp. 723–750. [90]	625	41.2	50.4%	Vets MH/SUD	Other health service—health service (veterans)	USA	- Multiple-Problem Screening Inventory (MPSI) subscales (of consideration—(l) confused thinking, (n) memory loss)
Bousman, C. A., Twamley, E. W., Vella, L., Gale, M., Norman, S. B., Judd, P., … Heaton, R. K. (2010). Homelessness and neuropsychological impairment: preliminary analysis of adults entering outpatient psychiatric treatment. *Journal of Nervous & Mental Disease*, *198*(11), 790–794. [91]	50	43	56.0%	MH	Mental Health/SUD Treatment Service—Health service (A & D/Psych)	USA	- Action Fluency (verbs) - Category Fluency (animals) - Controlled Oral Word Association Test (COWAT) (FAS) - Hopkins Verbal Learning Test-R (HVLT-R) - Wechsler Adult Intelligence Scale 3rdEd (WAIS-III) Symbol Search - Wechsler Adult Intelligence Scale 3rdEd (WAIS-III) Digit Symbol - Wide Range Achievement Test 4thEd (WRAT-IV)
Bremner, A. J., Duke, P. J., Nelson, H. E., Pantelis, C., & Barnes, T. R. E. (1996). Cognitive function and duration of rooflessness in entrants to a hostel for homeless men. *British Journal of Psychiatry*, *169*(OCT.), 434–439. [92]	62	not stated	100.0%	men only	Homeless service with accommodation	UK	- Adult memory and Information Processing battery (Information Processing Task B) - Mini Mental State Examination (MMSE) - NART/NART-R - Raven’s Standard Progressive Matrices - Single or series of questions, e.g., “Have you ever had a blow to the head?”
Brenner, L. A., Hostetter, T. A., Barnes, S. M., Stearns-Yoder, K. A., Soberay, K. A., & Forster, J. E. (2017). Traumatic brain injury, psychiatric diagnoses, and suicide risk among Veterans seeking services related to homelessness. *Brain Injury*, *31*(13/14), 1731–1735. [85]	309	52.3	96.1%	Vets MH/SUD	Other health service—Health service (veterans)	USA	- OSU TBI-ID
Brocht, C., Sheldon, P., & Synovec, C. (2020). *A clinical description of strategies to address traumatic brain injury experienced by homeless patients at Baltimore’s medical respite program. [References]*. Work: Journal of Prevention, Assessment & Rehabilitation. Vol.65(2), 2020, pp. 311–320. [51]	NA	NA	NA	adults	Health service for homeless people	USA	- Allen Cognitive Level Screen (ACLS-5) - Montreal Cognitive Assessment (MoCA) - WHODAS - OSU TBI-ID
Brown, R. T., Kiely, D. K., Bharel, M. & Mitchell, S. L. (2012). Geriatric syndromes in older homeless adults. *JGIM: Journal of General Internal Medicine*, *27*(1), 16–22. [93]	247	56	80.2%	older people	Homeless service with accommodation	USA	- Mini Mental State Examination (MMSE)
Brown, R. T., Hemati, K., Riley, E. D., Lee, C. T., Ponath, C., Tieu, L., … Kushel, M. B. (2017). Geriatric Conditions in a Population-Based Sample of Older Homeless Adults. *Gerontologist*, *57*(4), 757–766. [94]	350	58	77.1%	older people	Homeless service with accommodation	USA	- Mini Mental State Examination (MMSE) - Trail Making Test-Part B
Buhrich, N., Hodder, T., & Teesson, M. (2000). Prevalence of cognitive impairment among homeless people in inner Sydney. *Psychiatric Services*, *51*(4), 520–521. [95]	204	42.6	76.0%	men only	Homeless service with accommodation	Australia	- Mini Mental State Examination (MMSE)
Bymaster, A., Chung, J., Banke, A., Choi, H. J., & Laird, C. (2017). A Pediatric Profile of a Homeless Patient in San Jose, California. *Journal of Health Care for the Poor & Underserved*, *28*(1), 582–595. [13]	127	48	68.5%	adults	Health service for homeless people—Homeless medical clinic	USA	- OSU TBI-ID
Caplan, B., Schutt, R. K., Turner, W. M., Goldfinger, S. M., & Seidman, L. J. (2006). *Change in neurocognition by housing type and substance abuse among formerly homeless seriously mentally ill persons. [References]*. Schizophrenia Research. Vol.83(1), 2006, pp. 77–86. [39]	112	not stated	71.2%	MH	Health service for homeless people—homeless health service	USA	- Auditory Continuous Performance Test (CPT) - WMS-R Logical Memory (delayed condition) - Wisconsin Card Sorting Test (WCST)
Castaneda, R., Lifshutz, H., Galanter, M., & Franco, H. (1993). *Age at onset of alcoholism as a predictor of homelessness and drinking severity*. Journal of Addictive Diseases. Vol.12(1), 1993, 65–77. [96]	50	43	100.0%	SUD	Mental Health/SUD Treatment Service—Health service (A & D/Psych)	USA	- Modified Mini Mental State (3MS) Examination - Trail Making Test-Part A - Trail Making Test-Part B
Chang, F. H., Helfrich, C. A., Coster, W. J., & Rogers, E. S. (2015). Factors associated with community participation among individuals who have experienced homelessness. *International Journal of Environmental Research and Public Health*, *12*(9), 11364–11378. [35]	120	48.76	61.7%	adults	General, mixed or unspecified homelessness support	USA	- Allen Cognitive Level Screen-2000 (ACLS)
Cotman, A., & Sandman, C. (1997). Cognitive deficits and their remediation in the homeless. *Journal of Cognitive Rehabilitation*, *15*(1), 16–23. [97]	24	30.6	54.2%	adults	Homeless service with accommodation—homelessness transitional accomm program	USA	- Benton Visual Retention Test (BVRT) administration A - California Verbal Learning Test (CVLT) trials 1–5 - Test of Variables of Attention (TOVA) - Wechsler Adult Intelligence Scale—Revised Spatial Span Subtest as a neurological instrument (WAIS-RNI) - Wechsler Memory Scale—Revised (WMS-R) verbal memory - Wechsler Memory Scale—Revised (WMS-R) visual memory - WMS-R Logical Memory (delayed condition)
Dams-O’Connor, K., Cantor, J. B., Brown, M., Dijkers, M. P., Spielman, L. A., & Gordon, W. A. (2014). Screening for Traumatic Brain Injury: Findings and Public Health Implications. *Journal of Head Trauma Rehabilitation*, *29*(6), 479–489. [52]	111	not stated	not stated	adults	other—diverse community settings	USA	- Brain Injury Screening Index (BISI)
Davidson, D. (2007). *Prefrontal cortical function in the adult chronically homeless*. Dissertation Abstracts International: Section B: The Sciences and Engineering. Vol.68(6-B), 2007, 4170. [98]	30	42	50.0%	adults	Homeless service with accommodation	USA	- The Burglar’s Story - Controlled Oral Word Association Test (COWAT) (FAS) - Iowa Gambling Test
Davidson, D., Chrosniak, L., Wanschura, P., & Flinn, J. (2014). Indications of Reduced Prefrontal Cortical Function in Chronically Homeless Adults. *Community Mental Health Journal*, *50*(5), 548–552. [30]	46	42	50.0%	adults	Homeless service with accommodation	USA	- The Burglar’s Story - Controlled Oral Word Association Test (COWAT) (FAS) - Iowa Gambling Test
Distefano, R. (2020). *Autonomy support in parents and young children experiencing homelessness: A mixed method approach*. Dissertation Abstracts International Section A: Humanities and Social Sciences. Vol.81(9-A), 2020. [99]	28	37.9	100.0%	adults	Homeless service with accommodation	USA	- Minnesota Executive Function Scale (MEFS) - NIH Toolbox Picture Vocabulary (PVT)
Duerksen, C. L. (1994). *Differences between domiciled and undomiciled men on measures of executive functioning* [ProQuest Dissertations Publishing]. [100]	100	28.77	6.0%	adults	Homeless service with accommodation	USA	- Bender Visual Motor Gestalt Test - Cookie Card Theft Test (CCTT) - Line Tracing Test - Porteus Maze Test - Rey-Osterrieth Complex Figure Test (RCFT) - Stroop Colour Word Test - Tinkertoy Test - Wechsler Adult Intelligence Scale -Revised (WAIS-R)
Durbin, A., Lunsky, Y., Nisenbaum, R., Hwang, S. W., O’Campo, P., & Stergiopoulos, V. (2018). Borderline Intellectual Functioning and Lifetime Duration of Homelessness among Homeless Adults with Mental Illness. *Healthcare Policy*, *14*(2), 40–46. [101]	172	not stated	65.7%	co-/multi-morbidity	Housing support program to people at risk/formerly homeless	Canada	- NART/NART-R
Fichter, M. M., & Quadflieg, N. (2005). Three-year course and outcome of mental illness in homeless men—A prospective longitudinal study based on a representative sample. *European Archives of Psychiatry and Clinical Neuroscience*, *255*(2), 111–120. [102]	185	45.3	100.0%	men only	General, mixed or unspecified homelessness support—Street outreach	Germany	- Mini Mental State Examination (MMSE)
Foulks, E. F., McCown, W. G., Duckworth, M., & Sutker, P. B. (1990). Neuropsychological Testing of Homeless Mentally Ill Veterans. *Psychiatric Services*, *41*(6), 672–674. [103]	30	42.2	100.0%	MH	Homeless support without accommodation—homeless outreach	USA	- Porteus Maze Test - Stroop Colour Word Test - Wechsler Adult Intelligence Scale -Revised (WAIS-R)
Gabrielian, S., Bromley, E., Hellemann, G. S., Kern, R. S., Goldenson, N. I., Danley, M. E., & Young, A. S. (2015). Factors affecting exits from homelessness among persons with serious mental illness and substance use disorders. *Journal of Clinical Psychiatry*, *76*(4), e469–e476. [31]	36	52.6	100.0%	Vets MH/SUD	Homeless service with accommodation—Residential rehabilitation program for homeless adults	USA	- Hopkins Verbal Learning Test-R (HVLT-R) - Letter-Number Span (LNS) - Symbol Digit Modalities Test (SDMT)
Gabrielian, S., Bromley, E., Hamilton, A. B., Vu, V. T., Alexandrino, A., Jr., Koosis, E., & Young, A. S. (2019). *Problem solving skills and deficits among homeless veterans with serious mental illness.*. American Journal of Orthopsychiatry. Vol.89(2), 2019, pp. 287–295. [33]	40	52.3	87.5%	co-/multi-morbidity	Housing support program to people at risk/formerly homeless—Housing support (VA)	USA	- Category Fluency (animals) - Hopkins Verbal Learning Test-R (HVLT-R) - Symbol Digit Modalities Test (SDMT)
Gargaro, J., Gerber, G. J., & Nir, P. (2016). Brain Injury in Persons With Serious Mental Illness Who Have a History of Chronic Homelessness: Could This Impact How Services Are Delivered? *Canadian Journal of Community Mental Health*, *35*(2), 69–77. [14]	48	43.4	69.0%	MH	Health service for homeless people—MH Assertive Community Treatment Team servicing people who are homeless	Canada	- OSU TBI-ID
Gash, J. (2010). *Examining the relationship between spiritual resources, self-efficacy, life attitudes, cognition, and personal characteristics of homeless African American women* Wayne State University]. ccm. [73]	159	45.18	0.0%	women only	Homeless service with accommodation	USA	- Mini Mental State Examination (MMSE)
Gicas, K. M., Vila-Rodriguez, F., Paquet, K., Barr, A. M., Procyshyn, R. M., Lang, D. J., … Thornton, A. E. (2014). Neurocognitive profiles of marginally housed persons with comorbid substance dependence, viral infection, and psychiatric illness. *Journal of clinical and experimental neuropsychology*, *36*(10), 1009–1022. [104]	249	43.5%	?	adults	General, mixed or unspecified homelessness support—Hotel Study (observational study of people homeless and marginally housed)	Canada	- Cambridge Neuropsychological Test Automated Battery (CANTAB) Intra-Dimensional Extra-Dimensional subtest - Cambridge Neuropsychological Test Automated Battery (CANTAB) A-prime score from the Rapid Visual Information Processing subtest - Hopkins Verbal Learning Test-R (HVLT-R) - Iowa Gambling Test - Stroop Colour Word Test - Wechsler Test of Adult Reading (WTAR)
Gicas, K. M., Jones, A. A., Panenka, W. J., Giesbrecht, C., Lang, D. J., Vila-Rodriguez, F., … Honer, W. G. (2019). *Cognitive profiles and associated structural brain networks in a multimorbid sample of marginalized adults*. PLoS ONE. Vol.14(6), 2019, ArtID e0218201. [104]	208	42.6%	82.4%	adults	General, mixed or unspecified homelessness support	Canada	- Cambridge Neuropsychological Test Automated Battery (CANTAB) Intra-Dimensional Extra-Dimensional subtest - Cambridge Neuropsychological Test Automated Battery (CANTAB) A-prime score from the Rapid Visual Information Processing subtest - Hopkins Verbal Learning Test-R (HVLT-R) - Iowa Gambling Test - Stroop Colour Word Test - Wechsler Test of Adult Reading (WTAR)
Gicas, K. M., Thornton, A. E., Waclawik, K., Wang, N., Jones, A. A., Panenka, W. J., … Honer, W. G. (2019). Volumes of the Hippocampal Formation Differentiate Component Processes of Memory in a Community Sample of Homeless and Marginally Housed Persons. *Archives of clinical neuropsychology: the official journal of the National Academy of Neuropsychologists*, *34*(4), 548–562. [105]	227	40.9	79.0%	adults	General, mixed or unspecified homelessness support—Hotel Study (observational study of people homeless and marginally housed)	Canada	- Cambridge Neuropsychological Test Automated Battery (CANTAB) Intra-Dimensional Extra-Dimensional subtest - Cambridge Neuropsychological Test Automated Battery (CANTAB) A-prime score from the Rapid Visual Information Processing subtest - Hopkins Verbal Learning Test-R (HVLT-R) - Iowa Gambling Test - Stroop Colour Word Test - Wechsler Test of Adult Reading (WTAR)
Gilchrist, G., & Morrison, D. S. (2005). Prevalence of alcohol related brain damage among homeless hostel dwellers in Glasgow. *European Journal of Public Health*, *15*(6), 587–588. [10]	266	53	89.0%	adults	Homeless service with accommodation	UK (Scotland)	- Addenbrooke’s Cognitive Examination (ACE) - Battery to determine ARBD (ACE score < 88, plus positive screen for Fast Alcohol Screening Test (FAST) or CAGE Questionnaire, and Leeds Dependence Questionnaire)
Gonzalez, E. A., Dieter, J. N., Natale, R. A., & Tanner, S. L. (2001). Neuropsychological evaluation of higher functioning homeless persons: a comparison of an abbreviated test battery to the mini-mental state exam. *Journal of Nervous & Mental Disease*, *189*(3), 176–181. [54]	60	39.8	60.0%	adults	Health service for homeless people—medical service for homeless people	USA	- Aphasia Screening test (AST) - Mini Mental State Examination (MMSE) - Trail Making Test-Part A - Trail Making Test-Part B - WAIS-R Block design - WAIS-R Digit Symbol
Gouveia, L., Massanganhe, H., Mandlate, F., Mabunda, D., Fumo, W., Mocumbi, A. O., & de Jesus Mari, J. (2017). Family reintegration of homeless in Maputo and Matola: a descriptive study. *Int J Ment Health Syst*, *11*, 25. [106]	71	37.83	93.0%	MH	Mental Health/SUD Treatment Service—psychiatric hospital	Mozambique	- Mini International Neuropsychiatric Interview (MINI)
Heckert, U., Andrade, L., Alves, M. J. M. & Martins, C. (1999). Lifetime prevalence of mental disorders among homeless people in a southeast city in Brazil. *Eur Arch Psychiatry Clin Neurosci, 249*: 150–155. [107]	83	44	85.5%	adults	General, mixed or unspecified homelessness support	Brazil	- Mini Mental State Examination (MMSE)
Hegerty, S. M. (2010). *The neuropsychological functioning of men residing in a homeless shelter*. Dissertation Abstracts International: Section B: The Sciences and Engineering. Vol.70(8-B), 2010, 5197. [15]	51	45	100.0%	men only	Homeless service with accommodation	USA	- Boston Naming Test (BNT) - Connors’ Continuous Performance Test (CPT-II) - Dean–Woodcock Sensory Motor Battery (D-WSMB) Motor Functioning (Gait and Station, Romberg, Finger Tapping, Grip Strength) - Dean–Woodcock Sensory Motor Battery (D-WSMB) Sensory Functioning (Object Identification, Finger Identification) - Delis–Kaplan Executive Function System (D-KEFS) Verbal Fluency - Delis–Kaplan Executive Function System (D-KEFS) Tower - Delis–Kaplan Executive Function System (D-KEFS) Trail Making - Frontal Systems Behaviour Scale (FrSBe) - Grooved Pegboard Test - Rey Complex Figure Test (RCFT) copy trial - Rey Complex Figure Test (RCFT) immediate recall - Rey Complex Figure Test (RCFT) delayed recall - Wechsler Abbreviated Scale of Intelligence (WASI) - WAIS-III Digit Span - WAIS-III Digit Symbol-Coding - WAIS-III Letter-Number Sequencing - Wechsler Test of Adult Reading (WTAR) - Wide Range Assessment of Memory and Learning—2nd Ed (WRAML2) Verbal Memory - Wide Range Assessment of Memory and Learning—2nd Ed (WRAML2) Visual Memory - Wide Range Assessment of Memory and Learning—2nd Ed (WRAML2) Screening Memory - Single or series of questions, e.g., “Have you ever had a blow to the head?”
Henwood, B. F., Lahey, J., Rhoades, H., Pitts, D. B., Pynoos, J., & Brown, R. T. (2019). Geriatric Conditions Among Formerly Homeless Older Adults Living in Permanent Supportive Housing. *Journal of General Internal Medicine*, *34*(6), 802–803. [79]	237	41	87.0%	older people	Housing support program to people at risk/formerly homeless—permanent supportive housing agencies	USA	- Mini Mental State Examination (MMSE) - Trail Making Test-Part A - Trail Making Test-Part B
Hurstak, E., Johnson, J. K., Tieu, L., Guzman, D., Ponath, C., Lee, C. T., … Kushel, M. (2017). Factors associated with cognitive impairment in a cohort of older homeless adults: Results from the HOPE HOME study. *Drug and Alcohol Dependence*, *178*, 562–570. [108]	350	58.8	76.7%	older people	General, mixed or unspecified homelessness support	USA	- Modified Mini Mental State (3MS) Examination - Trail Making Test-Part B
Hux, K., Schneider, T., & Bennett, K. (2009). Screening for traumatic brain injury. *Brain Injury*, *23*(1), 8–14. [109]	240	39.93	14.0%	adults	Homeless service with accommodation	USA	- HELPS
Hwang, S. W., Colantonio, A., Chiu, S., Tolomiczenko, G., Kiss, A., Cowan, L., … Levinson, W. (2008). The effect of traumatic brain injury on the health of homeless people. *CMAJ: Canadian Medical Association Journal*, *179*(8), 779–784. [16]	904	71.7	66.5%	adults	Homeless service with accommodation	Canada	- Single or series of questions, e.g., “Have you ever had a blow to the head?”
Joyce, D. P., & Limbos, M. (2009). Identification of cognitive impairment and mental illness in elderly homeless men: Before and after access to primary health care. *Canadian Family Physician*, *55*(11), 1110–1111.e1116. [110]	29	51.8	100.0%	older people	Homeless service with accommodation	Canada	- Mini Mental State Examination (MMSE)
Keigher, S. M., & Greenblatt, S. (1992). Housing emergencies and the etiology of homelessness among the urban elderly. *Gerontologist*, *32*(4), 457–465. [111]	115	21.5	57.63%	older people	General, mixed homelessness—	USA	- Short Portable Mental Status Questionnaire (SPMSQ)
Keyser, D. R., Mathiesen, S. G. (2010). Co-occurring Disorders and Learning Difficulties: Client Perspectives From Two Community-Based Programs. *Psychiatric Services*, *61*(8), 841–844. [112]	37	41.2	52.5%	MH	Mental Health/SUD Treatment Service—mental health community services recovery centre/clubhouse	USA	- Learning Needs Screening Tool (LNST)
Lafferty, B. (2014). *Suicide risk in homeless veterans with traumatic brain injury* University of Pennsylvania]. ccm. [17]	122	43	Not stated	vets	General, mixed or unspecified homelessness support—National Center for Homelessness Among Veterans & 3x VAs	USA	- OSU TBI-ID - TBI-4
Llerena, K., Gabrielian, S., & Green, M. F. (2018). Clinical and cognitive correlates of unsheltered status in homeless persons with psychotic disorders. *Schizophrenia Research*, *197*, 421–427. [113]	76	not stated	94.7%	MH	General, mixed or unspecified homelessness support—supported housing waitlist	USA	- The MATRICS Consensus Cognitive Battery (MCCB)
Lo, P. C. (2001). *Cognitive functioning in a homeless population: The relationship of multiple traumatic brain injuries to neuropsychological test scores*. Dissertation Abstracts International: Section B: The Sciences and Engineering. Vol.62(3-B), 2001, 1586. [18]	128	52.3	64.1%	MH	Other health service—neuropsychology clinic	USA	- Boston Naming Test (BNT) - Colour Trails Test - Controlled Oral Word Association Test (COWAT) (FAS) - Grooved Pegboard Test - Neuropsychological Assessment Questionnaire - Rey-Osterrieth Complex Figure Design - Ruff Figural Fluency Test - Stroop Colour Word Test - Trail Making Test-Part A - Trail Making Test-Part B - Wechsler Adult Intelligence Scale 3rd Ed (WAIS-III) - Wechsler Memory Scale 3rdEd (WMS-III) - Wisconsin Card Sorting Test (WCST)
Lovisi, G. M., Mann, A. H., Coutinho, E., & Morgado, A. F. (2003). Mental illness in an adult sample admitted to public hostels in the Rio de Janeiro metropolitan area, Brazil. *Social Psychiatry and Psychiatric Epidemiology*, *38*(9), 493–498. [114]	319	NA	75.8%	adults	Homeless service with accommodation	Brazil	- Composite International Diagnostic Interview (CIDI1.1)
Mackelprang, J. L., Harpin, S. B., Grubenhoff, J. A., & Rivara, F. P. (2014). Adverse Outcomes Among Homeless Adolescents and Young Adults Who Report a History of Traumatic Brain Injury. *American Journal of Public Health*, *104*(10), 1986–1992. [115]	2732	56	36.7%	young people	General, mixed or unspecified homelessness support	USA	- The Wilder Homelessness Study survey (TBI data)
Mahmood, Z., Vella, L., Maye, J. E., Keller, A. V., Van Patten, R., Clark, J. M. R., & Twamley, E. W. (2021). Rates of Cognitive and Functional Impairments Among Sheltered Adults Experiencing Homelessness. *Psychiatric Services*, appips202000065. [49]	100	58	81.0%	adults	Homeless service with accommodation	USA	- Montreal Cognitive Assessment (MoCA) - University of California, San Diego, Performance Based Skills Assessment–Brief (UPSA-B) - WASI Matrix Reasoning subtest - WASI Vocabulary subtest - WAIS-IV Coding - Wide Range Achievement Test 4thEd (WRAT-IV)
Manthorpe, J., Samsi, K., Joly, L., Crane, M., Gage, H., Bowling, A., & Nilforooshan, R. (2019). Service provision for older homeless people with memory problems: a mixed-methods study. *NIHR Journals Library. Health Services and Delivery Research*, *2*, 2. [42]	42	42.6	87.1%	Older people	Homeless service with accommodation	UK	- Montreal Cognitive Assessment (MoCA) - Six-Item Cognitive Impairment Test (6-CIT) - Interview with case manager/key worker/support staff re whether they believe the person has memory problems/can live independently
Medalia, A., Herlands, T., & Baginsky, C. (2003). Rehab Rounds: Cognitive Remediation in the Supportive Housing Setting. *Psychiatric Services*, *54*(9), 1219–1220. [116]	12	48	not stated	adults	General, mixed homelessness—learning centre/supportive housing facility for people who are homeless and have mental illness	USA	- Cognitive Stability Index
Medalia, A., Saperstein, A. M., Huang, Y., Lee, S., & Ronan, E. J. (2017). Cognitive Skills Training for Homeless Transition-Age Youth: Feasibility and Pilot Efficacy of a Community Based Randomized Controlled Trial. *Journal of Nervous & Mental Disease*, *205*(11), 859–866. [117]	91	not stated	46.5%	young people	Housing support program to people at risk/formerly homeless—youth	USA	- California Verbal Learning Test (CVLT-II) immediate free recall - California Verbal Learning Test (CVLT-II) long delay free recall - Delis–Kaplan Executive Function System (D-KEFS) Tower - Delis–Kaplan Executive Function System (D-KEFS) Trail Making Number-Letter Switching - Delis–Kaplan Executive Function System (D-KEFS) Verbal Fluency-Letter Fluency - Delis–Kaplan Executive Function System (D-KEFS) Trail Making Visual Scanning - Delis–Kaplan Executive Function System (D-KEFS) Trail Making Number Sequencing - Delis–Kaplan Executive Function System (D-KEFS) Trail Making Letter Sequencing - WAIS-IV Digit Span - WAIS-IV Symbol Search - WAIS-IV Arithmetic - Wechsler Memory Scale 4thEd (WMS-IV) Logical Memory I - Wechsler Memory Scale 4thEd (WMS-IV) Logical Memory II
Mejia-Lancheros, C., Lachaud, J., Stergiopoulos, V., Matheson, F. I., Nisenbaum, R., O’Campo, P., & Hwang, S. W. (2020). Effect of Housing First on violence-related traumatic brain injury in adults with experiences of homelessness and mental illness: findings from the at Home/Chez Soi randomised trial, Toronto site. *BMJ Open*, *10*(12), Article e038443. [118]	381	43	68.0%	MH	Housing support program to people at risk/formerly homeless—housing first support program	Canada	- Single or series of questions, e.g., “Have you ever had a blow to the head?”
Monn, A. R. (2016). *Intergenerational processess in homeless families linking parent executive function, parenting quality, and child executive function*. Dissertation Abstracts International: Section B: The Sciences and Engineering. Vol.77(6-B(E)), 2016. [119]	105	48.76	5.5%	adults	Homeless service with accommodation	USA	- Go-NoGo - Peabody Picture Vocabulary Test, 4th Ed (PVT-IV) - Tower of London task (TOL) - WAIS-III Digit Span - WAIS-III Matrix Reasoning
Munoz, M., Vazquez, C., Koegel, P., Sanz, J., & Burnam, M. A. (1998). Differential patterns of mental disorders among the homeless in Madrid (Spain) and Los Angeles (USA). *Social Psychiatry and Psychiatric Epidemiology*, *33*(10), 514–520. [120]	1825	30.6	80.0%	adults	General, mixed or unspecified homelessness support	Spain/USA	- Mini Mental State Examination (MMSE)
Nikoo, N., Motamed, M., Nikoo, M. A., Strehlau, V., Neilson, E., Saddicha, S. & Krausz, M. (2011). Chronic Physical Health Conditions among Homeless. *Journal of Health Disparities Research and Practice, 8*(1), 81–97. [121]	500	not stated	59.8%	adults	Homeless service with accommodation	Canada	- National Survey of Homeless Assistance Providers and Clients (NSHAPC)—Health Chapter (Self Report) - National Survey of Homeless Assistance Providers and Clients (NSHAPC)—Health Chapter (Self-report head injury with resultant knock out or at least dizziness, confusion, or disorientation)
Nikoo, M., Gadermann, A., To, M. J., Krausz, M., Hwang, S. W., & Palepu, A. (2017). Incidence and Associated Risk Factors of Traumatic Brain Injury in a Cohort of Homeless and Vulnerably Housed Adults in 3 Canadian Cities. *Journal of Head Trauma Rehabilitation*, *32*(4), E19–E26. [122]	825	42	67.6%	adults	General, mixed or General, mixed or unspecified homelessness support (HHiT study—homeless and vulnerably housed (recently homeless) people)	Canada	- Single or series of questions, e.g., “Have you ever had a blow to the head?”
Nishio, A., Yamamoto, M., Horita, R., Sado, T., Ueki, H., Watanabe, T., … Shioiri, T. (2015). Prevalence of mental illness, cognitive disability, and their overlap among the homeless in Nagoya, Japan. *PLoS ONE [Electronic Resource]*, *10*(9), Article 0138052. [123]	114	42	93.0%	adults	General, mixed or unspecified homelessness support	Japan	- WAIS-III Digit Span - WAIS-III Digit Symbol-Coding - Wechsler Adult Intelligence Scale 3rdEd (WAIS-III) Picture completion - Wechsler Adult Intelligence Scale 3rdEd (WAIS-III) Information
Nishio, A., Horita, R., Sado, T., Mizutani, S., Watanabe, T., Uehara, R., & Yamamoto, M. (2017). Causes of homelessness prevalence: Relationship between homelessness and disability. *Psychiatry and Clinical Neurosciences*, *71*(3), 180–188. [124]	114	37.9	93.0%	adults	Other—social welfare agency	Japan	- WAIS-III Digit Span - WAIS-III Digit Symbol-Coding - Wechsler Adult Intelligence Scale 3rdEd (WAIS-III) Picture completion - Wechsler Adult Intelligence Scale 3rdEd (WAIS-III) Information
Oakes, P. D. & R. C. (2008). Intellectual disability in homeless adults: A prevalence study. *Journal of Intellectual Disabilities*, *12*(4), 325–334. [125]	50	28.77	66.0%	adults	Other health service—PCT-run general practice	UK	- Adaptive Behaviour Assessment Scale (ABAS) - WASI Vocabulary subtest
Oddy, M., Moir, J. F., Fortescue, D., & Chadwick, S. (2012). The prevalence of traumatic brain injury in the homeless community in a UK city [Multicenter Study Research Support, Non-U.S. Gov’t Review]. *Brain Injury*, *26*(9), 1058–1064. [126]	100	not stated	75.0%	adults	Homeless service with accommodation	UK	- Single or series of questions, e.g., “Have you ever had a blow to the head?”
Okamura, T., Awata, S., Ito, K., Takiwaki, K., Matoba, Y., Niikawa, H., … Takeshima, T. (2017). Elderly men in Tokyo homeless shelters who are suspected of having cognitive impairment. *Psychogeriatrics: The Official Journal of the Japanese Psychogeriatric Society*, *17*(3), 206–207. [127]	51	45.3	100.0%	older people	Homeless service with accommodation	Japan	- Mini Mental State Examination (MMSE)
Palladino, B., Montgomery, A. E., Sommers, M., & Fargo, J. D. (2017). Risk of Suicide Among Veterans with Traumatic Brain Injury Experiencing Homelessness. *Journal of Military & Veterans’ Health*, *25*(1), 34–38. [128]	103	42.2	100.0%	vets	General, mixed or unspecified homelessness support—Homeless Support (VA)	USA	- OSU TBI-ID - TBI-4
Pluck, G., Kwang-Hyuk, L., David, R., Macleod, D. C., Spence, S. A., & Parks, R. W. (2011). Neurobehavioural and cognitive function is linked to childhood trauma in homeless adults. *British Journal of Clinical Psychology*, *50*(1), 33–45. https://doi.org/10.1348/014466510X490253 [27]	55	52.6	80.0%	adults	Homeless service with accommodation	UK	- Frontal Systems Behaviour Scale (FrSBe) - Wechsler Abbreviated Scale of Intelligence (WASI) - Single or series of questions, e.g., “Have you ever had a blow to the head?”
Pluck, G., Lee, K. H., David, R., Spence, S. A., & Parks, R. W. (2012). Neuropsychological and Cognitive Performance of Homeless Adults. *Canadian Journal of Behavioural Science-Revue Canadienne Des Sciences Du Comportement*, *44*(1), 9–15. [129]	80	52.3	83.8%	MH	General, mixed or unspecified homelessness support	UK	- WASI Vocabulary subtest - Wechsler Memory Scale 4thEd (WMS-IV) - Wechsler Test of Adult Reading (WTAR)
Pluck, G., Nakakarumai, M., & Sato, Y. (2015). Homelessness and cognitive impairment: An exploratory study in Tokyo, Japan [Article]. *East Asian Archives of Psychiatry*, *25*(3), 122–127. [130]	16	43.4	100.0%	men only	Homeless service with accommodation—homeless service (skills development)	Japan	- Japanese Adult Reading Test - Mini Mental State Examination (MMSE) - Wisconsin Card Sorting Test (WCST)
Pluck, G., Barajas, B. M., Hernandez-Rodriguez, J. L., & Martínez, M. A. (2020). Language ability and adult homelessness. *International Journal of Language & Communication Disorders*, *55*(3), 332–344. [131]	17	45.18	94.1%	adults	General, mixed or unspecified homelessness support—homeless service	Ecuador	- Mini Mental State Examination (MMSE) - Word Accentuation Test (Spanish) - HELPS
Raphael-Greenfield, E. (2012). Assessing Executive and Community Functioning Among Homeless Persons with Substance Use Disorders Using the Executive Function Performance Test. *Occupational Therapy International*, *19*(3), 135–143. [132]	60	43.5%	91.5%	SUD	Housing support program to people at risk/formerly homeless—housing first support program	USA	- Executive Function Performance Test (EFPT)
Rogans-Watson, R., Shulman, C., Lewer, D., Armstrong, M., & Hudson, B. (2020). Premature frailty, geriatric conditions and multimorbidity among people experiencing homelessness: a cross-sectional observational study in a London hostel. *Housing Care and Support*, *23*(3–4), 77–91. [133]	33	42.6%	91.0%	adults	Homeless service with accommodation—homeless hotel	UK	- Rowland universal dementia assessment scale (RUDAS)
Rogoz, A., & Burke, D. (2016). Older people experiencing homelessness show marked impairment on tests of frontal lobe function. *International Journal of Geriatric Psychiatry*, *31*(3), 240–246. [134]	171	40.9	84.2%	older people	General, mixed or unspecified homelessness support	Australia	- Clock Drawing test - Controlled Oral Word Association Test (COWAT) (FAS) - Mini Mental State Examination (MMSE) - Self-Report of cognitive function and memory—interview only, no formal tool - Trail Making Test-Part B
Rohde, P., Noell, J., & Ochs, L. (1999). IQ scores among homeless older adolescents: characteristics of intellectual performance and associations with psychosocial functioning. *Journal of Adolescence*, *22*(3), 319–328. [135]	50	53	50.0%	young people	Homeless support without accommodation—homeless outreach program	USA	- Wechsler Adult Intelligence Scale -Revised (WAIS-R)
Roy, S., Svoboda, T., Issacs, B., Budin, R., Sidhu, A., Biss, R. K., … Connelly, J. O. (2020). Examining the Cognitive and Mental Health Related Disability Rates Among the Homeless: Policy Implications of Changing the Definition of Disability in Ontario. *Canadian Psychology*, *61*(2), 118–126. [136]	1872	39.8	69.0%	adults	Homeless service with accommodation	Canada	- CASE MANAGER ASSESSMENT: Rapid Assessment of Residential Support (RARS)
Russell, L. M., Devore, M. D., Barnes, S. M., Forster, J. E., Hostetter, T. A., Montgomery, A. E., … Brenner, L. A. (2013). Challenges associated with screening for traumatic brain injury among US veterans seeking homeless services. *American Journal of Public Health*, *103 Suppl 2*, S211–212. [19]	678	37.83	94.7%	vets	Other health service—Health service (veterans)	USA	- OSU TBI-ID - TBI-4
San Agustin, M., Cohen, P., Rubin, D., Cleary, S. D., Erickson, C. J. & Allen, J. K. (1999). The Montefiore Community Children’s Project: A controlled study of cognitive and emotional problems of homeless mothers and children. *Journal of Urban Health: Bulletin of the New York Academy of Medicine, 76*(1), 39–50. [137]	82	44	0.0%	women only	Homeless service with accommodation	USA	- Peabody Picture Vocabulary Test—Revised (PVT-R) - Raven’s Standard Progressive Matrices
Saperstein, A. M., Lee, S., Ronan, E. J., Seeman, R. S., & Medalia, A. (2014). Cognitive deficit and mental health in homeless transition-age youth. *Pediatrics*, *134*(1), e138–145. [34]	67	45	51.5%	young people	General, mixed or unspecified homelessness support—Homeless service (youth)	USA	- California Verbal Learning Test (CVLT-II) immediate free recall - California Verbal Learning Test (CVLT-II) long delay free recall - Delis–Kaplan Executive Function System (D-KEFS) Tower - Delis–Kaplan Executive Function System (D-KEFS) Trail Making Number-Letter Switching - Delis–Kaplan Executive Function System (D-KEFS) Verbal Fluency-Letter Fluency - Delis–Kaplan Executive Function System (D-KEFS) Trail Making Visual Scanning - Delis–Kaplan Executive Function System (D-KEFS) Trail Making Number Sequencing - Delis–Kaplan Executive Function System (D-KEFS) Trail Making Letter Sequencing - WAIS-III Arithmetic - WAIS-III Digit Span - WAIS-III Digit Symbol-Coding - Wechsler Adult Intelligence Scale 3rdEd (WAIS-III) Symbol Search - Wechsler Memory Scale 4thEd (WMS-IV) Logical Memory I - Wechsler Memory Scale 4thEd (WMS-IV) Logical Memory II - Wechsler Test of Adult Reading (WTAR)
Schmitt, T., Thornton, A. E., Rawtaer, I., Barr, A. M., Gicas, K. M., Lang, D. J., … Panenka, W. J. (2017). Traumatic Brain Injury in a Community-Based Cohort of Homeless and Vulnerably Housed Individuals. *Journal of Neurotrauma*, *34*(23), 3301–3310. [138]	283	41	77.1%	adults	Housing support program to people at risk/formerly homeless—single room occupancy housing	Canada	- Cambridge Neuropsychological Test Automated Battery (CANTAB) Intra-Dimensional Extra-Dimensional subtest - Cambridge Neuropsychological Test Automated Battery (CANTAB) A-prime score from the Rapid Visual Information Processing subtest - Hopkins Verbal Learning Test-R (HVLT-R) - Wechsler Test of Adult Reading (WTAR) - Iowa Gambling Test - Stroop Colour Word Test - Wechsler Test of Adult Reading (WTAR) - Single or series of questions, e.g., “Have you ever had a blow to the head?”
Schutt, R. K., Seidman, L. J., Caplan, B., Martsinkiv, A., & Goldfinger, S. M. (2007). The role of neurocognition and social context in predicting community functioning among formerly homeless seriously mentally ill persons. *Schizophrenia Bulletin*, *33*(6), 1388–1396. [139]	112	58.8	71.2%	MH	Homeless service with accommodation	USA	- auditory Continuous Performance Test (CPT) - WMS-R Logical Memory (delayed condition) - Wisconsin Card Sorting Test (WCST)
Seidman, L. J., Caplan, B. B., Tolomiczenko, G. S., Turner, W., Penk, W., Schutt, R. K., & Goldfinger, S. M. (1997). Neuropsychological Function in Homeless Mentally Ill Individuals. *The Journal of Nervous & Mental Disease*, *185*(1), 3–12. [140]	116	39.93	72.4%	MH	Homeless service with accommodation	USA	- auditory Continuous Performance Test (CPT) - Benton Line Orientation - Finger Tapping Test - Motor Sequencing (non-preferred hand)—Luria Manual Hand Positions Test - Motor Sequencing (preferred hand)—Luria Manual Hand Positions Test - Porteus Maze Test - Visual Verbal Total Misses - WAIS-R Digit Span - WAIS-R Block design - WAIS-R Digit Symbol - WAIS-R Vocabulary - Wechsler Memory Scale—Revised (WMS-R) verbal memory - WMS-R Logical Memory (immediate) - WMS-R Logical Memory (delayed condition) - Wisconsin Card Sorting Test (WCST)
Seidman, L. J., Schutt, R. K., Caplan, B., Tolomiczenko, G. S., Turner, W. M., & Goldfinger, S. M. (2003). *The Effect of Housing Interventions on Neuropsychological Functioning Among Homeless Persons With Mental Illness*. Psychiatric Services. Vol.54(6), 2003, 905–908. [38]	112	71.7	71.2%	MH	Homeless service with accommodation	USA	- auditory Continuous Performance Test (CPT) - Benton Line Orientation - Finger Tapping Test - Motor Sequencing (non-preferred hand)—Luria Manual Hand Positions Test - Motor Sequencing (preferred hand)—Luria Manual Hand Positions Test - Porteus Maze Test - Visual Verbal Total Misses - WAIS-R Digit Span - WAIS-R Block design - WAIS-R Digit Symbol - WAIS-R Vocabulary - Wechsler Memory Scale—Revised (WMS-R) verbal memory - Wide Range Achievement Test 4thEd (WRAT-R) Arithmetic - Wide Range Achievement Test 4thEd (WRAT-R) Reading - Wide Range Achievement Test 4thEd (WRAT-R) Spelling - WMS-R Logical Memory (immediate) - WMS-R Logical Memory (delayed condition) - Wisconsin Card Sorting Test (WCST)
Soar, K., Papaioannou, G., & Dawkins, L. (2016). Alcohol Gel Ingestion Among Homeless Eastern and Central Europeans in London: Assessing the Effects on Cognitive Functioning and Psychological Health. *Substance Use & Misuse*, *51*(10), 1274–1282. [141]	45	51.8	100.0%	CALD/BME	Homeless support without accommodation—homelessness resource centre	UK	- Prospective and Retrospective Memory Questionnaire (PRMQ) - WAIS-III Block design - WAIS-III Digit Span
Solliday-McRoy, C. L. (2002). *Neuropsychological functioning of homeless men in shelter: An exploratory study*. Dissertation Abstracts International: Section B: The Sciences and Engineering. Vol.63(4-B), 2002, 2087. [142]	75	21.5	100.0%	men only	Homeless service with accommodation	USA	- Neurobehavioural cognitive Status Examination (Cognistat) - Rey Auditory Verbal Learning Test (RAVLT) - Rey-Osterrieth Complex Figure Test (RCFT) - Tower of Hanoi - Wechsler Abbreviated Scale of Intelligence (WASI) - WAIS-III Digit Span - Woodcock Johnson Psychoeducational Battery-Revised: Tests of Achievement (WJ-R ACH) (Letter-Word Identification and Passage Comprehension Subtests)
Solliday-McRoy, C., Campbell, T. C., Melchert, T. P., Young, T. J., & Cisler, R. A. (2004). Neuropsychological functioning of homeless men. *Journal of Nervous & Mental Disease*, *192*(7), 471–478. [29]	90	41.2	100.0%	men only	Homeless service with accommodation	USA	- Neurobehavioural cognitive Status Examination (Cognistat) - Rey Auditory Verbal Learning Test (RAVLT) - Rey-Osterrieth Complex Figure Test (RCFT) - Wechsler Abbreviated Scale of Intelligence (WASI) - Woodcock Johnson Psychoeducational Battery-Revised: Tests of Achievement (WJ-R ACH) (Letter-Word Identification and Passage Comprehension Subtests)
Song, M. J., Nikoo, M., Choi, F., Schutz, C. G., Jang, K., & Krausz, R. M. (2018). Childhood Trauma and Lifetime Traumatic Brain Injury Among Individuals Who Are Homeless. *Journal of Head Trauma Rehabilitation*, *33*(3), 185–190. [20]	487	43	57.7%	adults	General, mixed or unspecified homelessness support (British Columbia Health of the Homeless Survey)	Canada	- Single or series of questions, e.g., “Have you ever had a blow to the head?”
Souza, A. M., Tsai, J. H. C., Pike, K. C., Martin, F., & McCurry, S. M. (2020). Cognition, Health, and Social Support of Formerly Homeless Older Adults in Permanent Supportive Housing. *Innovation in Aging*, *4*(1), 1–9. [143]	53	not stated	86.8%	older people	Housing support program to people at risk/formerly homeless—permanent supportive housing agencies	USA	- Mini-Cog - Patient Reported Outcomes Measure Information System (PROMIS) measure: Cognition
Stergiopoulos, V., Cusi, A., Bekele, T., Skosireva, A., Latimer, E., Schutz, C., … Rourke, S. B. (2015). Neurocognitive impairment in a large sample of homeless adults with mental illness. *Acta Psychiatrica Scandinavica*, *131*(4), 256–268. [32]	1500	52.3	67.0%	MH	Housing support program to people at risk/formerly homeless—housing first support program	Canada	- Trail Making Test-Part A - Trail Making Test-Part B - Hopkins Verbal Learning Test-R (HVLT-R) - WAIS-R Digit Symbol
Synovec, C. E. (2018). Occupational Therapy in Health Care Agencies Serving Adults Experiencing Homelessness: Outcomes of a Pilot Model. *Occupational Therapy in Health Care Agencies Serving Adults Experiencing Homelessness: Outcomes of a Pilot Model*, 1–1. [144]	172	NA	73.0%	adults	Health service for homeless people	USA	- Assessment of Motor and Process Skills (AMPS) - Allen Cognitive Level Screen (ACLS-5) - Executive Function Performance Test (EFPT) - Montreal Cognitive Assessment (MoCA)
Synovec, C. E. (2020). Evaluating Cognitive Impairment and Its Relation to Function in a Population of Individuals Who Are Homeless. *Occupational Therapy in Mental Health*, *36*(4), 330–352. [11]	172	56	73.0%	adults	Health service for homeless people	USA	- Assessment of Motor and Process Skills (AMPS) - Allen Cognitive Level Screen (ACLS-5) - Executive Function Performance Test (EFPT) - Montreal Cognitive Assessment (MoCA)
Synovec, C. E., & Berry, S. (2020). Addressing brain injury in health care for the homeless settings: A pilot model for provider training. *Work: Journal of Prevention, Assessment & Rehabilitation. Vol.65*(2), 2020, pp. 285–296. [21]	172	58	73.0%	adults	Health service for homeless people	USA	- OSU TBI-ID
Teesson, M., & Buhrich, N. (1993). Prevalence of Cognitive Impairment Among Homeless Men in a Shelter in Australia. *Psychiatric Services*, *44*(12), 1187–1189. [145]	56	42.6	100.0%	men only	Homeless service with accommodation	Australia	- Mini Mental State Examination (MMSE)
Thompson, A., Richardson, P., Pirmohamed, M., & Owens, L. (2020). Alcohol-related brain injury: An unrecognized problem in acute medicine. *Alcohol*, *88*, 49–53. [146]	1276	48	69.4%	SUD	Other health service—hospital	UK	- Montreal Cognitive Assessment (MoCA) - Combination: Identified High Risk for ARBI (through YES to either 3x hosp in 12 months; 2x hosp in 30 days; family concern re cognition) Plus MoCA < 23
To, M. J., O’Brien, K., Palepu, A., Hubley, A. M., Farrell, S., Aubry, T., … Hwang, S. W. (2015). Healthcare Utilization, Legal Incidents, and Victimization Following Traumatic Brain Injury in Homeless and Vulnerably Housed Individuals: A Prospective Cohort Study. *Journal of Head Trauma Rehabilitation*, *30*(4), 270–276. [22]	1181	not stated	66.2%	adults	Homeless service with accommodation	Canada	- Single or series of questions, e.g., “Have you ever had a blow to the head?”
Topolovec-Vranic, J., Ennis, N., Howatt, M., Ouchterlony, D., Michalak, A., Masanic, C., … Cusimano, M. D. (2014). Traumatic brain injury among men in an urban homeless shelter: observational study of rates and mechanisms of injury. *CMAJ open*, *2*(2), E69–76. [147]	111	43	100.0%	men only	Homeless service with accommodation	Canada	- Brain Injury Screening Questionnaire (BISQ)
Topolovec-Vranic, J., Schuler, A., Gozdzik, A., Somers, J., Bourque, P. E., Frankish, C. J., … Hwang, S. W. (2017). The high burden of traumatic brain injury and comorbidities amongst homeless adults with mental illness. *Journal of Psychiatric Research*, *87*, 53–60. [23]	2088	48.76	67.6%	MH	Housing support program to people at risk/formerly homeless—Homelessness program (At Home/Chez Soi)	Canada	- Single or series of questions, e.g., “Have you ever had a blow to the head?”
Twamley, E. W., Hays, C. C., Van Patten, R., Seewald, P. M., Orff, H. J., Depp, C. A., … Jak, A. J. (2019). Neurocognition, psychiatric symptoms, and lifetime homelessness among veterans with a history of traumatic brain injury. *Psychiatry Research*, *271*, 167–170. [148]	32	30.6	100.0%	vets	Other health service—medical records from veterans referred for neuropsych testing following TBI	USA	- California Verbal Learning Test (CVLT-II) trials 1–5 - California Verbal Learning Test (CVLT-II) long delay free recall - Delis–Kaplan Executive Function System (D-KEFS) Colour Word Inhibition Test - Delis–Kaplan Executive Function System (D-KEFS) Trail Making Number-Letter Switching - Delis–Kaplan Executive Function System (D-KEFS) Verbal Fluency-Category Fluency - Delis–Kaplan Executive Function System (D-KEFS) Verbal Fluency-Category Switching - Delis–Kaplan Executive Function System (D-KEFS) Verbal Fluency-Letter Fluency - Rey Complex Figure Test (RCFT) copy trial - Rey Complex Figure Test (RCFT) delayed recall - WAIS-IV Digit Span - WAIS-IV Coding - Wide Range Achievement Test 4thEd (WRAT-IV) - Wisconsin Card Sorting Test (WCST)
Valera, E. M., & Berenbaum, H. (2003). *Brain injury in battered women.* Journal of Consulting and Clinical Psychology. Vol.71(4), 2003, 797–804. [24]	99	not stated	0.0%	women only	General, mixed or unspecified homelessness support—programs targeting women leaving or experiencing violence	USA	- California Verbal Learning Test (CVLT) long delay free recall - California Verbal Learning Test (CVLT) trials 1–5 - Trail Making Test-Part A - Trail Making Test-Part B - WAIS-R Digit Span - Single or series of questions, e.g., “Have you ever had a blow to the head?”
Van Straaten, B., Rodenburg, G., Van der Laan, J., Boersma, S. N., Wolf, J. R., & Van de Mheen, D. (2017). Self-reported care needs of Dutch homeless people with and without a suspected intellectual disability: a 1.5-year follow-up study. *Health & Social Care in the Community*, *25*(1), 123–136. [40]	336	42	84.6%	adults	General, mixed or unspecified homelessness support	Netherlands	- Hayes Ability Screening Index (HASI)
Vella, L. (2015). *Cognitive assessment of the sheltered homeless*. Dissertation Abstracts International: Section B: The Sciences and Engineering. Vol.75(12-B(E)), 2015. [41]	100	42	81.0%	adults	Homeless service with accommodation	USA	- Montreal Cognitive Assessment (MoCA) - Wechsler Abbreviated Scale of Intelligence (WASI) - WAIS-IV Coding - Wide Range Achievement Test 4thEd (WRAT-IV) - Interview with case manager/key worker/support staff re whether they believe the person has memory problems/can live independently
Vila-Rodriguez, F., Panenka, W. J., Lang, D. J., Thornton, A. E., Vertinsky, T., Wong, H., … Honer, W. G. (2013). The hotel study: Multimorbidity in a community sample living in marginal housing. *American Journal of Psychiatry*, *170*(12), 1413–1422. [149]	293	37.9	76.8%	adults	General, mixed or unspecified homelessness support—Hotel Study (observational study of people homeless and marginally housed)	Canada	- Cambridge Neuropsychological Test Automated Battery (CANTAB) Intra-Dimensional Extra-Dimensional subtest - Cambridge Neuropsychological Test Automated Battery (CANTAB) A-prime score from the Rapid Visual Information Processing subtest - Hopkins Verbal Learning Test-R (HVLT-R) - Iowa Gambling Test - Stroop Colour Word Test - Wechsler Test of Adult Reading (WTAR) - Single or series of questions, e.g., “Have you ever had a blow to the head?”
Waclawik, K., Jones, A. A., Barbic, S. P., Gicas, K. M., O’Connor, T. A., Smith, G. N., … Thornton, A. E. (2019). Cognitive Impairment in Marginally Housed Youth: Prevalence and Risk Factors. *Frontiers in Public Health*, *7*, 270. [150]	101	28.77	75.0%	young people	Homeless service with accommodation	Canada	- Cambridge Neuropsychological Test Automated Battery (CANTAB) Intra-Dimensional Extra-Dimensional subtest - Cambridge Neuropsychological Test Automated Battery (CANTAB) A-prime score from the Rapid Visual Information Processing subtest - Hopkins Verbal Learning Test-R (HVLT-R) - Iowa Gambling Test - Stroop Colour Word Test - Wechsler Test of Adult Reading (WTAR)
White, P., Townsend, C., Lakhani, A., Cullen, J., Bishara, J., & White, A. (2019). The Prevalence of Cognitive Impairment among People Attending a Homeless Service in Far North Queensland with a Majority Aboriginal and/or Torres Strait Islander People. *Australian Psychologist*, *54*(3), 193–201. [36]	60	not stated	67.0%	indigenous	General, mixed or unspecified homelessness support—homeless service (veterans)	Australia	- Kimberley Indigenous Cognitive Assessment - OSU TBI-ID
Yim, L. C., Leung, H. C., Chan, W. C., Lam, M. H., & Lim, V. W. (2015). Prevalence of Mental Illness among Homeless People in Hong Kong. *PLoS ONE [Electronic Resource]*, *10*(10), e0140940. [151]	79	45.3	93.7%	adults	General, mixed or unspecified homelessness support—NGO Homelessness services	Hong Kong	- Mini Mental State Examination (MMSE)
Zhou, L. W., Panenka, W. J., Jones, A. A., Gicas, K. M., Thornton, A. E., Heran, M. K. S., … Field, T. S. (2019). Prevalence and Risk Factors of Brain Infarcts and Associations With Cognitive Performance in Tenants of Marginal Housing. *Journal of the American Heart Association*, *8*(13), 1–11. [152]	228	42.2	77.0%	adults	Homeless service with accommodation (Crisis/shelter)—Homelessness service (SRO Hotels)	Canada	- Cambridge Neuropsychological Test Automated Battery (CANTAB) Intra-Dimensional Extra-Dimensional subtest - Cambridge Neuropsychological Test Automated Battery (CANTAB) A-prime score from the Rapid Visual Information Processing subtest - Hopkins Verbal Learning Test-R (HVLT-R) - Iowa Gambling Test - Stroop Colour Word Test - Wechsler Test of Adult Reading (WTAR)
Zlotnick, C., Fischer, P. J., & Agnew, J. (1995). Perceptuomotor function of homeless males in alcohol rehabilitation. *Journal of Substance Abuse*, *7*(2), 235–244. [153]	76	52.6	100.0%	SUD	Mental Health/SUD Treatment Service—Health service (A & D/Psych)	USA	- Finger Tapping Test - Mini Mental State Examination (MMSE) - Purdue Pegboard - Simple Visual Reaction Time (SVRT) - Trail Making Test-Part A - Trail Making Test-Part B - WAIS-R Digit Symbol - WAIS-R Vocabulary

## Appendix C

**Table A3:** Complete list of screening instruments.

Individual Instruments (Including Each Version)	Times Described
**Cognitive Screens**	
Action Fluency (verbs)	1
Adaptive Behaviour Assessment Scale (ABAS)	1
Addenbrooke’s Cognitive Examination (ACE)	1
Addenbrooke’s Cognitive Examination III (ACE-III)	1
Adult memory and Information Processing battery (Information Processing Task B)	1
Assessment of Motor and Process Skills (AMPS)	2
Allen Cognitive Level Screen-2000 (ACLS)	1
Allen Cognitive Level Screen (ACLS-5)	3
Aphasia Screening test (AST)	1
Bender Visual Motor Gestalt Test	1
Benton Line Orientation	2
Benton Visual Retention Test (BVRT) administration A	1
Boston Naming Test (BNT)	2
The Burglar’s Story	2
California Verbal Learning Test (CVLT) long delay free recall	1
California Verbal Learning Test (CVLT) trials 1–5	2
California Verbal Learning Test (CVLT)	1
California Verbal Learning Test (CVLT-II) trials 1–5	1
California Verbal Learning Test (CVLT-II) immediate free recall	2
California Verbal Learning Test (CVLT-II) long delay free recall	3
Cambridge Neuropsychological Test Automated Battery (CANTAB) Intra-Dimensional Extra-Dimensional subtest	7
Cambridge Neuropsychological Test Automated Battery (CANTAB) A-prime score from the Rapid Visual Information Processing subtest	7
Category Fluency (animals)	2
Clock Drawing test	1
Cognitive Stability Index	1
Colour Trails Test	1
Composite International Diagnostic Interview (CIDI; version 1.1)	1
Cookie Card Theft Test (CCTT)	1
Connors’ Continuous Performance Test (CPT-II)	2
Controlled Oral Word Association Test (COWAT) (FAS)	6
auditory Continuous Performance Test (CPT)	4
Dean–Woodcock Sensory Motor Battery (D-WSMB) Motor Functioning (Gait and Station, Romberg, Finger Tapping, Grip Strength)	1
Dean–Woodcock Sensory Motor Battery (D-WSMB) Sensory Functioning (Object Identification, Finger Identification)	1
Delis–Kaplan Executive Function System (D-KEFS) Colour Word Inhibition Test	2
Delis–Kaplan Executive Function System (D-KEFS) Verbal Fluency	1
Delis–Kaplan Executive Function System (D-KEFS) Tower	1
Delis–Kaplan Executive Function System (D-KEFS) Trail Making	4
Delis–Kaplan Executive Function System (D-KEFS) Trail Making motor speed (test #5)	1
Delis–Kaplan Executive Function System (D-KEFS) Trail Making Number-Letter Switching	4
Delis–Kaplan Executive Function System (D-KEFS) Verbal Fluency-Category Fluency	2
Delis–Kaplan Executive Function System (D-KEFS) Verbal Fluency-Category Switching	2
Delis–Kaplan Executive Function System (D-KEFS) Verbal Fluency-Letter Fluency	4
Delis–Kaplan Executive Function System (D-KEFS) Trail Making Visual Scanning	3
Delis–Kaplan Executive Function System (D-KEFS) Trail Making Number Sequencing	3
Delis–Kaplan Executive Function System (D-KEFS) Trail Making Letter Sequencing	3
Executive Function Performance Test (EFPT)	3
Finger Tapping Test	3
Frontal Systems Behaviour Scale (FrSBe)	2
Go-NoGo	1
Grooved Pegboard Test	2
Hayes Ability Screening Index (HASI)	1
Hopkins Verbal Learning Test-R (HVLT-R)	12
Iowa Gambling Test	8
Japanese Adult Reading Test	1
Kimberley Indigenous Cognitive Assessment	1
Learning Needs Screening Tool (LNST)	1
Letter-Number Span (LNS)	1
Line Tracing Test	1
The MATRICS Consensus Cognitive Battery (MCCB)	1
Mini International Neuropsychiatric Interview (MINI)	1
Mini-Cog	1
Mini Mental State Examination (MMSE)	20
Minnesota Executive Function Scale (MEFS; Carlson & Zelatzp, 2014)	1
Modified Mini Mental State (3MS) Examination	2
Montreal Cognitive Assessment (MoCA)	7
Motor Sequencing (non-preferred hand)—Luria Manual Hand Positions Test	2
Motor Sequencing (preferred hand)—Luria Manual Hand Positions Test	2
Multiple-Problem Screening Inventory (MPSI) subscales (of consideration—(l) confused thinking, (n) memory loss)	1
NART/NART-R	2
Neurobehavioural cognitive Status Examination (Cognistat)	2
Neuropsychological Assessment Questionnaire	1
NIH Toolbox Picture Vocabulary (PVT)	1
Peabody Picture Vocabulary Test—Revised (PVT-R)	1
Peabody Picture Vocabulary Test, 4th Ed (PVT-IV)	1
Patient Reported Outcomes Measure Information System (PROMIS) measure: Cognition	1
Porteus Maze Test	4
Purdue Pegboard	1
Prospective and Retrospective Memory Questionnaire (PRMQ)	1
Raven’s Standard Progressive Matrices	3
Rey Auditory Verbal Learning Test (RAVLT)	2
Rey Complex Figure Test (RCFT) copy trial	2
Rey Complex Figure Test (RCFT) immediate recall	1
Rey Complex Figure Test (RCFT) delayed recall	2
Rey-Osterrieth Complex Figure Design	1
Rey-Osterrieth Complex Figure Test (RCFT)	3
Repeatable Battery for the Assessment of Neuropsychological Status (RBANS)	2
Rowland universal dementia assessment scale (RUDAS)	1
Ruff Figural Fluency Test	1
Self-Report of cognitive function and memory—interview only, no formal tool	2
Short Portable Mental Status Questionnaire (SPMSQ)	1
Simple Visual Reaction Time (SVRT)	1
Six-Item Cognitive Impairment Test (6-CIT)	1
Stroop Colour Word Test	10
Symbol Digit Modalities Test (SDMT)	2
Test of Variables of Attention (TOVA)	1
Tinkertoy Test	1
Tower of Hanoi	1
Tower of London task (TOL)	1
Trail Making Test-Part A	8
Trail Making Test-Part B	11
University of California, San Diego, Performance-Based Skills Assessment–Brief (UPSA-B)	1
Visual Verbal Total Misses	2
Wechsler Abbreviated Scale of Intelligence (WASI)	6
Wechsler Abbreviated Scale of Intelligence (WASI) Matrix Reasoning subtest	1
Wechsler Abbreviated Scale of Intelligence (WASI) Vocabulary subtest	3
Wechsler Adult Intelligence Scale 3rd Ed (WAIS-III)	1
Wechsler Adult Intelligence Scale 3rd Ed (WAIS-III) Arithmetic	1
Wechsler Adult Intelligence Scale 3rd Ed (WAIS-III) Block design	1
Wechsler Adult Intelligence Scale 3rd Ed (WAIS-III) Digit Span	7
Wechsler Adult Intelligence Scale 3rd Ed (WAIS-III) Digit Symbol-Coding	4
Wechsler Adult Intelligence Scale 3rd Ed (WAIS-III) Letter-Number Sequencing	1
Wechsler Adult Intelligence Scale 3rd Ed (WAIS-III) Matrix Reasoning	1
Wechsler Adult Intelligence Scale 3rdEd (WAIS-III) Symbol Search	2
Wechsler Adult Intelligence Scale 3rdEd (WAIS-III) Digit Symbol	1
Wechsler Adult Intelligence Scale 3rdEd (WAIS-III) Picture completion	2
Wechsler Adult Intelligence Scale 3rdEd (WAIS-III) Information	2
Wechsler Adult Intelligence Scale 4thEd (WAIS-IV)	1
WAIS-IV Digit Span	2
WAIS-IV Coding	3
WAIS-IV Symbol Search	1
WAIS-IV Arithmetic	1
WAIS-R Digit Span	3
WAIS-R Block design	3
WAIS-R Digit Symbol	5
WAIS-R Vocabulary	3
Wechsler Adult Intelligence Scale -Revised (WAIS-R)	3
Wechsler Adult Intelligence Scale—Revised Spatial Span Subtest as a neurological instrument (WAIS-RNI)	1
Wechsler Memory Scale 3rdEd (WMS-III)	1
Wechsler Memory Scale 4thEd (WMS-IV)	2
Wechsler Memory Scale 4thEd (WMS-IV) Logical Memory I	2
Wechsler Memory Scale 4thEd (WMS-IV) Logical Memory II	2
Wechsler Memory Scale—Revised (WMS-R) Logical Memory Immediate Recall	2
Wechsler Memory Scale—Revised (WMS-R) Logical Memory Delayed Recall	5
Wechsler Memory Scale—Revised (WMS-R) Verbal Memory	3
Wechsler Memory Scale—Revised (WMS-R) Visual Memory	1
Wechsler Test of Adult Reading (WTAR)	10
WHODAS	1
Wide Range Achievement Test 4thEd (WRAT-R)—Spelling	1
Wide Range Achievement Test 4thEd (WRAT-R)—Reading	1
Wide Range Achievement Test 4thEd (WRAT-R)—Arithmetic	1
Wide Range Achievement Test 4thEd (WRAT-IV)	5
Wide Range Assessment of Memory and Learning—2nd Ed (WRAML2) Verbal Memory	1
Wide Range Assessment of Memory and Learning—2nd Ed (WRAML2) Visual Memory	1
Wide Range Assessment of Memory and Learning—2nd Ed (WRAML2) Screening Memory	1
Wisconsin Card Sorting Test (WCST)	8
Woodcock Johnson Psychoeducational Battery-Revised: Tests of Achievement (WJ-R ACH) (Letter-Word Identification and Passage Comprehension Subtests)	2
Word Accentuation Test (Spanish)	1
National Survey of Homeless Assistance Providers and Clients (NSHAPC)—Health Chapter (Self Report)	1
CASE MANAGER ASSESSMENT: Rapid Assessment of Residential Support (RARS)	1
CASE MANAGER ASSESSMENT: Interview with case manager/key worker/support staff re whether they believe the person has memory problems/can live independently	2
Combination: Identified High Risk for ARBI (through YES to either 3x hosp in 12 months; 2x hosp in 30 days; family concern re cognition) Plus MoCA < 23	1
**Brain Injury Screens**	
Brain Injury Screening Index (BISI)	2
Brain Injury Screening Questionnaire (BISQ)	2
HELPS	2
OSU TBI-ID	10
TBI-4	3
Single or series of questions, e.g., “Have you ever had a blow to the head?”	14
National Survey of Homeless Assistance Providers and Clients (NSHAPC)—Health Chapter (Self-report Head injury with Knock out, dizziness, confusion, or disorientation)	1
